# Best Practices for Implementing Electronic Care Records in Adult Social Care: Rapid Scoping Review

**DOI:** 10.2196/60107

**Published:** 2025-02-14

**Authors:** Martha Snow, Wagner Silva-Ribeiro, Mary Baginsky, Sonya Di Giorgio, Nicola Farrelly, Cath Larkins, Karen Poole, Nicole Steils, Joanne Westwood, Juliette Malley

**Affiliations:** 1 Care Policy and Evaluation Centre London School of Economics and Political Science (LSE) London United Kingdom; 2 NIHR Health & Social Care Workforce Research Unit King's College London London United Kingdom; 3 Libraries & Collections King's College London London United Kingdom; 4 School of Health, Social Work and Sport University of Central Lancashire Preston, Lancashire United Kingdom

**Keywords:** digital care records, adult social care, digitization, domiciliary care, care homes, electronic care records, PRISMA

## Abstract

**Background:**

In the past decade, the use of digital or electronic records in social care has risen worldwide, capturing key information for service delivery. The COVID-19 pandemic accelerated digitization in health and social care. For example, the UK government created a fund specifically for adult social care provider organizations to adopt digital social care records. These developments offer valuable learning opportunities for implementing digital care records in adult social care settings.

**Objective:**

This rapid scoping review aimed to understand what is known about the implementation of digital care records in adult social care and how implementation varies across use cases, settings, and broader contexts.

**Methods:**

A scoping review methodology was used, with amendments made to enable a rapid review. Comprehensive searches based on the concepts of digital care records, social care, and interoperability were conducted across the MEDLINE, EmCare, Web of Science Core Collection, HMIC Health Management Information Consortium, Social Policy and Practice, and Social Services Abstracts databases. Studies published between 2018 and 2023 in English were included. One reviewer screened titles and abstracts, while 2 reviewers extracted data. Thematic analysis mapped findings against the nonadoption, abandonment, scale-up, spread, and sustainability (NASSS) framework.

**Results:**

Our search identified 2499 references. After screening titles and abstracts, 71 records were selected for full-text review, resulting in 31 references from 29 studies. Studies originated from 11 countries, including 1 multicountry study, with the United Kingdom being the most represented (10/29, 34%). Studies were most often conducted in nursing homes or facilities (7/29, 24%) with older people as the target population (6/29, 21%). Health records were the most investigated record type (12/29, 41%). We identified 45 facilitators and 102 barriers to digital care record implementation across 28 studies, spanning 6 of the 7 NASSS framework domains and aligning with 5 overarching themes that require greater active management regarding implementation. Intended or actual implementation outcomes were reported in 17 (59%) of the 29 studies.

**Conclusions:**

The findings suggest that implementation is complex due to a lack of consensus on what digital care records and expected outcomes and impacts should look like. The literature often lacks clear definitions and robust study designs. To be successful, implementation should consider complexity, while studies should use robust frameworks and mixed methods or quantitative designs where appropriate. Future research should define the target population, gather data on carer or service user experiences, and focus on digital care records specifically used in social care.

## Introduction

### Background

The demand for adult social care is vast. Global demographic changes throughout the 20th century have led to substantial population aging, decreased mortality and communicable diseases, and increased chronic noncommunicable diseases. Consequently, more adults and older people have long-term care needs, particularly in high-income countries where the epidemiological transition began earlier [1]. Technology has been proposed to help manage this increasing demand in health and social care by improving efficiency, care quality, and effectiveness [2-4]. Digital care records are one such innovation in adult social care.

In this paper, adult social care refers to long-term, aged, or disability care, including care homes; support in the home; domiciliary care (eg, personal care, practical tasks, and crisis support); community-based support such as inclusive arts programs; and social relationships that aim to keep people independent, active, and living well.

The use of digital care records has increased across various adult social care settings and countries since 2012 [5]. These records capture key information for service delivery, including individuals’ characteristics, the care they receive, and how they respond to it. They monitor service users and track service delivery, supporting care planning, medication, and assessments [6-10]. In addition, they serve administrative purposes [8,11,12], support compliance with data documentation regulations [13], and inform care delivery decisions [14,15]. Different terms are used to describe digital care records in social care. In the United Kingdom, the term digital social care records (DSCRs) is common. In North America, parts of Europe, and Australia, terms such as electronic health records [16-20], electronic patient records [6], or electronic medical records [9,19] are often used. Digital care records can be part of health information exchange initiatives, which facilitate data sharing across health and social care to improve care continuity and efficiency [16-20].

Despite the increasing use of digital care records, much of the existing literature focuses on their implementation in nursing homes or approaches the topic from a social work perspective, failing to capture the full scope of adult social care. One systematic review of electronic health records identified that health information exchange is facilitated by workflow integration and flexible organizational culture and impeded by incomplete data, inefficiency, and unfavorable market conditions [21]. Another review found that electronic health records support health outcomes, clinical documentation management, and decision-making [2]. To the best of our knowledge, the only previous review of DSCRs that looked at the benefits of implementation was a review by Greenstock [22]. This literature review highlighted improved documentation and health outcomes as well as increased collaboration and communication, efficiency, quality of care, client or family involvement, and risk management [22]. It identified less evidence regarding financial benefits and increased workforce satisfaction [22]. It is unclear how many benefits were realized versus anticipated [22]. A scoping review of electronic information systems in social care found that they can negatively affect social workers’ priorities and do not meet sector needs [23].

These reviews predate the COVID-19 pandemic, which accelerated digital system development in health and social care [24]. For example, the UK government injected funds during the COVID-19 pandemic to drive digitization and has since continued these efforts. A specific fund for adult social care provider organizations supports DSCR adoption, with the most recent government target of 80% adoption across adult social care provider organizations in England by March 2025.

### Objectives

The intensity of the activity discussed above presented an opportunity to learn about DSCR implementation and impact through evaluation. Considering recent developments, this rapid scoping review sought to assess what is known about DSCR implementation in adult social care settings and identify evidence gaps to inform a rapid evaluation of DSCR implementation. While this purpose has influenced decisions around the methods, such as a rapid approach and more intensive searching for UK literature, the review considers the international literature on DSCR implementation and draws out implications for an international audience.

We mapped our findings against the nonadoption, abandonment, scale-up, spread, and sustainability (NASSS) framework by Greenhalgh et al [25]. Designed in 2017 as an evidence-based, theory-informed, and pragmatic tool, it helps predict and evaluate the success of technology-supported health or social care programs. As it focuses on adoption, nonadoption, and abandonment of technologies as well as the challenges associated with the scale-up, spread, and maintenance of digital systems, it was deemed appropriate for capturing the field’s complexity. The framework was particularly useful during data analysis. Most of the literature retrieved identified large numbers of facilitators of and barriers to DSCR adoption. The NASSS framework helped to position these within an interrelated system and organize them in a way that could provide guidance in areas requiring active management of complexity. As the NASSS framework has been applied more often to health care settings, this review was also an opportunity to explore its value for technology adoption in social care.

## Methods

### Overview

The rapid scoping review followed the 6-stage framework outlined by Arksey and O’Malley [26], which was later refined by Levac et al [27] and the Joanna Briggs Institute [28]. Following the study by Tricco et al [29], we made some amendments to enable a rapid review. The review is reported in accordance with the PRISMA-ScR (Preferred Reporting Items for Systematic Reviews and Meta-Analyses extension for Scoping Reviews) guidelines (Multimedia Appendix 1) [30]. The search strategy is reported in accordance with the PRISMA-S (Preferred Reporting Items for Systematic reviews and Meta-Analyses literature search extension) checklist [31]. A protocol for this review was developed using the PRISMA-ScR and registered prospectively with the Open Science Framework on August 9, 2023 [32].

### Identifying the Research Question

We used the Joanna Briggs Institute’s population, concept, and context framework [28] to formulate the following scoping review questions: (1) What is known about the implementation of DSCRs in social care settings? and (2) How does implementation vary across use cases, social care settings, and the broader context? The subquestions were as follows:

- What DSCR is being used?
- What situation or setting is the DSCR being used in, and which actors are involved?
- What is the broader context within which DSCRs are being implemented or used?
- What is the use case for the DSCR, and what are the intended outcomes and benefits?
- How has the implementation of DSCRs been evaluated or researched, and what theoretical framings have been used?
- What are the intended or actual outcomes and benefits of DSCR implementation?
- What helps or gets in the way of the implementation of DSCRs?

### Identifying Relevant Studies

A librarian with experience in undertaking reviews (KP) designed the search in consultation with the research team. The search was undertaken between August 2, 2023, and August 11, 2023, by 2 librarians (KP and SDG) across MEDLINE (through Ovid; KP), EmCare (through Ovid; SDG), Web of Science Core Collection (Clarivate; KP), HMIC Health Management Information Consortium (through Ovid; KP), Social Policy and Practice (through Ovid; KP), and Social Services Abstracts (through ProQuest; SDG) databases.

The search strategy used 3 concepts: digital care records, social care, and interoperability. These concepts were combined in the search string as (Digital Care Records AND Social Care) OR (Social Care AND Interoperability). The interoperability concept was included, as it is central to policy narratives surrounding the implementation of DSCRs in England, with expectations that DSCRs will facilitate data sharing with general practitioners and hospitals. The initial search strategy was developed on MEDLINE (Ovid) by one of the librarians (KP) and run in each database by KP and SDG. Publications were limited to those published in or after 2018 until 2023. The results were limited to the English language. The databases were searched using keywords and controlled vocabulary (eg, Medical Subject Headings or Emtree) where appropriate and adapted according to the requirements of each database. The full search strategy for each database can be found in Multimedia Appendix 2.

There were 3466 results in total. The results were exported to EndNote (Clarivate), and 993 duplicates were removed following a structured process [33], leaving 2473 unique results. These were exported as a research information systems file to Covidence (Veritas Health Innovation Ltd) software [34] for title and abstract screening as well as for full-text review.

In addition, given the intention of informing an evaluation in the context of the English language, we searched key English websites to capture gray literature not identified through the databases. The chosen websites were the Local Government Association [35]; King’s Fund [36]; Social Care Institute of Excellence [37]; Centre for Care [38]; Digital Care Hub, formerly Digital Social Care [39]; and TEC Service Association [40]. Searches were also performed on Google, and we contacted experts identified through the review. From these searches and reference checking, 27 references were identified. Of these 27 references, 1 (4%) duplicate was removed, and 1 (4%) reference that reported results from a study already included was merged with the main reference. One reference recommended by an expert was also included. This resulted in 26 references retrieved through our gray literature search.

### Study Selection

We included studies that (1) took place within adult social care settings; (2) involved the implementation of a DSCR, which may be referred to by other labels, such as electronic care records and electronic information systems; (3) were carried out using any study design (eg, experimental, quasi-experimental, and observational, including quantitative and qualitative studies); and (4) were published from 2018 onward. This decision was made on the basis that existing reviews have captured the literature on DSCRs up until the end of 2017.

Following rapid review methodology guidance [29,41], all references retrieved from our search were screened by a reviewer with expertise in systematic reviews (WSR). Initial screening was based on titles and abstracts. References were selected for full-text review if they met our inclusion criteria or if it was unclear that they did. The same reviewer (WSR) performed the full-text review. A second reviewer, who is an expert in adult social care research (JM), cross-checked references that were excluded in this phase. Disagreement was discussed until a consensus was reached.

### Charting the Data

A data extraction template was developed by the team using Excel (Microsoft Corp). The form included key characteristics of included studies, such as the population, concept, context, study design, and methods, and key findings that were relevant to the review questions. In total, 2 reviewers (MS and WSR) performed the data extraction. Due to the heterogeneity of studies and following best practice, the extraction form was piloted and iteratively adapted through discussions between the 2 reviewers and a third reviewer (JM), who oversaw the extraction process.

During the data extraction, we discovered that 2 publications [42,43] reported results from the same study. Another publication [44] was a preprint version of 1 peer-reviewed article [45], which was also included in the review. All publications were included to ensure we used the information available, but to avoid duplication of information, we extracted information at the study level rather than the publication level.

### Collating, Reporting, and Summarizing the Results

There were several steps involved in collating and reporting the results. We first created a summary of the included studies, categorizing the papers according to relevant study characteristics, such as study design, population, context, methods of data collection and analysis, and theoretical perspectives. We then worked inductively to identify intended or actualized benefits and outcomes and barriers to and facilitators of implementation raised in the papers. Using thematic analysis, we compiled a descriptive overview of the unique barriers and facilitators identified in the papers, including frequency distributions.

We then used the NASSS framework as a sensitizing framework and worked deductively to ensure we had not missed anything of relevance to the NASSS domains. In this process, further barriers and facilitators were identified, and these were mapped alongside those identified from the inductive process to the NASSS domains and subdomains. Where a category was associated with >1 NASSS domain, it was mapped against the domain perceived as most affected.

To synthesize our findings, we then grouped the barriers and facilitators into themes capturing complex aspects of the adoption process. Complexity was determined using the NASSS framework, which defines implementation as simple (ie, few components and predictable), complicated (ie, many components but still largely predictable), or complex (ie, many components interacting in a dynamic and unpredictable way) [46]. The more complexity there is in the system, the less likely the technology is to achieve sustained adoption across the system, and the more likely it is to be abandoned [46]. The themes draw attention to areas that require greater active management with respect to implementation [25].

## Results

### Overview

**Figure 1:**
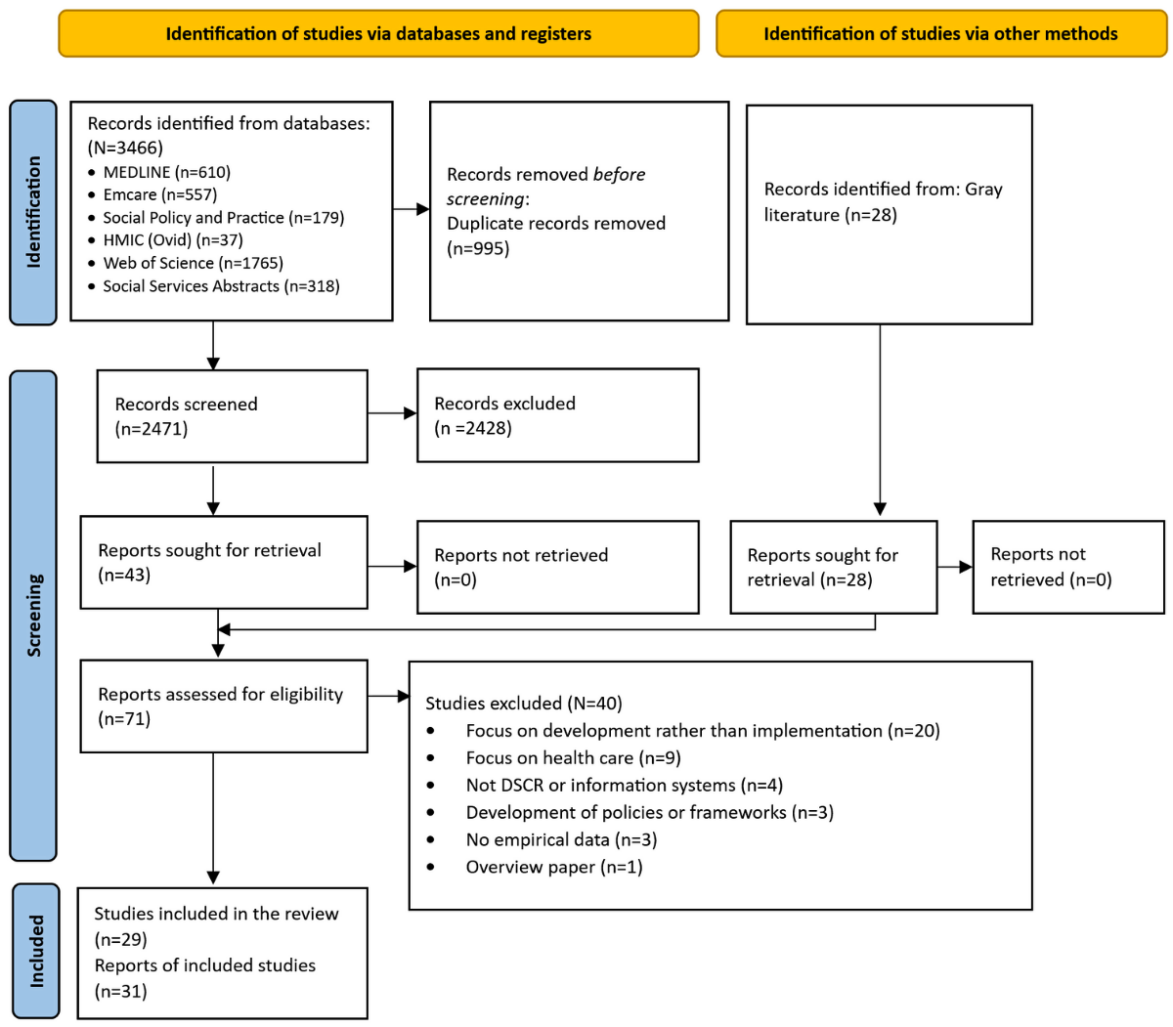
PRISMA (Preferred Reporting Items for Systematic Reviews and Meta-Analyses) 2020 flowchart. DSCR: digital social care record.

Our search resulted in 2471 references after duplicates were removed. An additional 28 references were identified through the gray literature search, resulting in 2499 references. After screening references based on titles and abstracts, 71 records were selected for full-text review, of which 31 references were included from 29 different studies (ie, 2 pairs of papers reported on the same studies). The article selection process and reasons for exclusion are presented in Figure 1 [47].

### Study Characteristics

As Table 1 presents, of the 29 studies, 10 (34%) were conducted in the United Kingdom—7 (24%) in England [3,7,10,20,48-50], 2 (7%) in Scotland [43,45], and 1 (3%) [51] in multiple UK countries. Of the 29 studies, 5 (17%) were conducted in the United States [9,14-16,18], 3 (10%) in Finland [52-54], 2 (7%) in Australia [11,13], 2 (7%) in Canada [19,55], and 2 (7%) in Sweden [12,56]. The remaining studies were conducted in Switzerland [8], Japan [57], Austria [17], and Italy [58] or involved multiple countries [6].

Studies in the United Kingdom were conducted in care homes (3/29, 10%) [43,45,48], health and social care provider organizations (4/29, 14%) [3,7,20,49], multiple social care settings (1/29, 3%) [51], councils (1/29, 3%) [50], and a continuing health care team (1/29, 3%) [10]. More than one-third of studies from other countries were conducted in nursing homes and facilities (7/29, 24%) [6,8,9,14,15,17,18]. Other settings included home care (5/29, 17%) [12,55-58], care homes (1/29, 3%) [13], long-term care facilities (1/29, 3%) [11], an acute care hospital and its neighboring long-term care home (1/29, 3%) [19], assisted living communities (1/29, 3%) [16], health centers in Finland (2/29, 7%) [52,53], and social care services (1/29, 3%) [54].

**Table 1:** Key characteristics of the included studies (N=29).

Characteristic		Studies, n (%)
**Design**		
	Cohort	1 (3)
	Cross-sectional	6 (21)
	Mixed methods	8 (28)
	Qualitative	14 (48)
**Country**		
	Australia	2 (7)
	Austria	1 (3)
	Canada	2 (7)
	Finland	3 (10)
	Italy	1 (3)
	Japan	1 (3)
	Sweden	2 (7)
	Switzerland	1 (3)
	United Kingdom	10 (35)
	United States	5 (17)
	Belgium, Czech Republic, and Spain	1 (3)
**Aim**		
	Barriers and facilitators	9 (31)
	Prevalence of use of DSCRs^a^	7 (24)
	Professionals’ perceptions about DSCRs	4 (14)
	Impact of DSCRs on professionals’ work	4 (14)
	Services’ readiness to implement DSCRs	3 (10)
	Strategies to improve DSCRs	1 (3)
	Impact of DSCRs on health outcomes	1 (3)
**Setting**		
	Assisted living community	1 (3)
	Care homes	4 (14)
	Continuing or community health care	3 (10)
	Councils	1 (3)
	Home care	5 (17)
	Multisector (ie, health and social care)	6 (21)
	Municipal social services	1 (3)
	Nursing homes or facilities	7 (24)
	Social care provider organizations	1 (3)
**Population**		
	Older people	6 (21)
	Adults with care needs	2 (7)
	People with dementia	1 (3)
	Not specified	20 (69)
**Respondents^b^**		
	Social care staff	10 (35)
	Health care staff	8 (28)
	Social care managers	8 (28)
	Health care managers	3 (10)
	Users or carers	2 (7)
	Regional stakeholders	2 (7)
	National stakeholders	1 (3)
	Technology providers or vendors	1 (3)
	Not specified	6 (21)
**Types of technology**		
	Health records	12 (41)
	Health and social care records	8 (28)
	Social care records	4 (14)
	Interoperability	4 (14)
	Not specified	1 (3)
**Theoretical framework used^b^**		
	The DeLone and McLean model of information systems success	1 (3)
	The Wang and Strong quality framework	1 (3)
	Activity theory	1 (3)
	Sociotechnical systems theory	1 (3)
	Computer-supported cooperative work	1 (3)
	Design thinking	1 (3)
	Nolan stage model	1 (3)
	Normalization process theory	2 (7)
	Implementation process framework	1 (3)
	Unified theory of acceptance and use of technology	1 (3)
	Not specified or applicable	20 (69)

^a^DSCR: digital social care record.

^b^Total is >100% because some studies collected information with different types of informants.

Studies investigated several different types of digital care records—from health information technology in general (4/29, 14%) [18,48,55,57] to electronic medical records or electronic health records specifically (12/29, 41%) [6,8,9,11,12, 14-18,53,56]. Some were specific systems commissioned by or developed for care providers, local authorities, or regions, such as the aged care ecosystem [13], the Edotto regional information system [58], CareFirst [10], the PASSsystem [51], or CareCentric [3], among others. Of the 29 studies, 4 (14%) focused on interoperability [10,19,20,57]. Studies can be grouped into those which aimed to (1) identify barriers or facilitators to the implementation of DSCRs and information exchange systems (9/29, 31%) [3,10,13,19,20,48-50,53], (2) assess the proportion and prevalence of services using DSCRs and information exchange systems and how these are being used (7/29, 24%) [14-18,45,56], (3) investigate how digital systems affect the work of care professionals or care providers (4/29, 14%) [7,11,51,54], (4) assess care professionals’ perceptions about the use of digital systems (4/29, 14%) [6,8,52,55], (5) map services’ readiness or maturity and care professionals’ capability to adopt DSCRs and information exchange systems (3/29, 10%) [9,12,42], (6) assess potential strategies to improving existing DSCRs or information exchange systems (1/29, 3%) [58], and (7) assess the impact of use of information communication technology (ICT) on health outcomes (1/29, 3%) [57].

To achieve these aims, most studies used a qualitative design (14/29, 48%) [3,6,7,11-13,17,48-50,52,53,56,58]. Of the 29 studies, 8 (28%) used mixed methods [9,10,19,20,45,51,54,55] and 7 (24%) used a quantitative design [8,14-16,18,43,57]. None of the studies that aimed to identify barriers or facilitators, to investigate how digital systems affect work routines, or to assess potential strategies to improve digital systems used quantitative methods. Among the studies that aimed to assess professionals’ perceptions of digital systems, only 1 was quantitative [8]. Of the studies that aimed to assess the proportion and prevalence of digital systems, most (4/29, 14%) used quantitative methods [14-16,18]. The study assessing the impact of ICT on health outcomes was also quantitative [57]. The studies that aimed to map services’ readiness and maturity varied between qualitative [12], quantitative [42], and mixed methods approaches [9].

Most studies (20/29, 69%) did not use a theoretical framework to interpret their results [3,8-10,14-17,19,42,45,48-52,54-57]. Of those that did, theories included normalization process theory (2/29, 7%) [20,53], sociotechnical systems theory (1/29, 3%) [6], the Nolan stage model (1/29, 3%) [18], an implementation process framework (1/29, 3%) [13], the DeLone and McLean model of information systems success and the Wang and Strong quality framework (1/29, 3%) [58], activity theory (1/29, 3%) [11], design thinking (1/29, 3%) [12], computer-supported cooperative work (1/29, 3%) [7], and the unified theory of acceptance and use of technology (1/29, 3%) [20].

A detailed list of the characteristics of all studies included in this review is provided in Multimedia Appendix 3 [3,6-20, 42-45,48-58].

### Summary of Facilitators of and Barriers to the Implementation of DSCRs

#### Overview

Of the 29 studies, 28 (97%) identified 45 facilitators of and 102 barriers to digital implementation. These were then coded into 32 categories that aligned with the NASSS framework domains: 18 contained facilitators and 24 contained barriers (the total is >32 because some categories contained both facilitators and barriers). The most frequent barriers were related to the digital system lacking interoperability, which was found in 10 of the 29 studies (34%). They also related to insufficient funding or financial incentives and high costs of implementation (9/29, 31%), and technology not matching the context of use (9/29, 31%). Most facilitators were associated with building interorganizational trust and collaborative relationships (5/29, 17%); adequate training (5/29, 17%); anticipating, frontloading, and resourcing the work required to clarify information governance (4/29, 14%); skillful leadership enhancing an organization’s digital readiness and capacity for change (4/29, 14%); and high usability of the digital system (4/29, 14%).

Regarding the NASSS framework domains, most facilitators were related to the organization (24/45, 53%). This was followed by the adopter system (8/45, 18%), the technology (6/45, 13%), the value proposition (4/45, 9%), the interaction between domains and adaptation over time (2/45, 4%), and the wider context (1/45, 2%). Most barriers were also related to the organization (52/102, 51%). This was followed by the technology (25/102, 24.5%), the wider context (14/102, 13.7%), the value proposition (6/102, 5.9%), the adopter system (4/102, 3.9%), and the interaction between domains and adaptation over time (1/102, 1%). No barriers or facilitators were related to the condition domain.

The categories containing facilitators and barriers were then organized into five broad themes: (1) the legal and institutional context for holding and sharing data and its effect on the ability and willingness to share data, (2) digital readiness and organizational capacity for change, (3) using and sharing recorded information within technical constraints, (4) alignment between care practices and digital recording practices, and (5) differences between what is expected and what is achievable with digital systems.

A summary of how barriers and facilitators identified in each study were mapped to categories, themes, and the NASSS framework domains and subdomains is provided in Multimedia Appendix 4 [3,6-20,42-45,48-56,58]. The 5 themes are summarized in greater detail in the subsequent sections.

#### Legal and Institutional Context for Holding and Sharing Data and Its Effect on Ability and Willingness to Share Data

A key challenge to DSCR implementation involved information governance concerns about holding and sharing data. These issues arose from vague legislation, market competition, conflicting priorities, poor internal and external coordination, and low cross-organizational trust. Building trust and adequately resourcing digital change facilitated implementation.

##### Commercial and Regulatory Context in Which Care Providers Operate

A total of 3 studies conducted in the United Kingdom [3,42,50] identified barriers related to market competition among social care provider organizations and digital suppliers and a lack of national regulation and standards. Private sector care providers were concerned that the commercial sensitivity of data could compromise their competitive advantage [42]. Vendor lock-in also occurred, as technology suppliers hesitated to share data with other suppliers [3].

The governance and ethics framework for social care data in the United Kingdom is less developed compared to that for National Health Service (NHS) data. There is no established system for the governance of care home data, which are held by private companies, care regulators, and health and social care provider organizations [42]. This context made data sharing challenging [42]. Despite regulatory progress, councils found new national frameworks inadequate on data and interoperability standards [50].

##### Interorganizational Trust and Relationships

In 5 studies [3,9,42,48,49], 4 of which were UK based, a lack of trust between providers and other organizations hampered information governance and data sharing. Clinical and health care partners were particularly reluctant to share data with social care [3,9,50] due to misunderstandings about their role and concerns about sharing information with staff who were not registered social workers [50]. Ownership of a large volume of patient data and responsibility for confidentiality also fostered a risk-averse attitude among general practitioners [3].

Four studies conducted in the United Kingdom [3,42,49,50] found that building trust and collaboration between organizations facilitated implementation. Care homes were more willing to share information when they had well-established relationships with local authorities [42]. Scotland’s regional “data safe havens,” led by trusted partners such as the NHS, academic institutions, and government agencies, represented a centralized approach to managing, storing, and handling access requests to health care data that encouraged relationships between health and social care provider organizations [42]. They were an example of data being handled respectfully, professionally, and securely [42]. Ambiguous governance frameworks necessitated clarifying information governance requirements and building mutual trust in systems. In the United Kingdom, local authority and provider staff needed to dedicate significant resources upfront to ensure safe data handling processes [3]. Setting up information sharing agreements that specified data flows between organizations could be intensive, involving unexpected time and effort that was often related to building relationships and engaging numerous actors with data sharing plans [3]. Undertaking this work early on in projects facilitated implementation, locating expertise and capacity, and building trust across organizations [3]. Leaders who fostered positive working relationships between decision makers facilitated shared priority setting [49], helping them to circumvent barriers stemming from organizational fragmentation [49].

##### Organizational Coordination to Clarify Information Governance

In 4 studies [3,9,42,49], a general lack of coordination hindered the clarification of information governance processes needed to implement digital systems. Divisions between and within organizations created siloed data systems, resulting in residents’ records being stored in different systems across multiple services [49]. Poor coordination was linked to information governance professionals, who managed personal data for single organizations, lacking the capacity to handle additional responsibilities for cross-organization information governance and data sharing [3]. This issue was compounded by provider leaders’ lack of understanding of information governance [3].

Lack of a shared, standardized understanding of information governance and data ownership across organizations also created confusion among staff. Nursing home leaders in the United States [9], for example, raised concerns about transparency and maintaining control of residents’ health data that were viewed as belonging to the patients, leading to fear of lawsuits regarding data sharing [9]. In the United Kingdom, there was a lack of shared understanding with confusion about consent, which was related to social care and local government starting from a different position to NHS partners when it came to information sharing [50].

While a lack of organizational coordination was a barrier to implementation, 4 studies [3,10,49,58] identified that prioritizing and adequately resourcing the work required to define information governance was a facilitator to data sharing and in turn improved service quality. In the implementation of information systems across home care services in Italy [58], agreements could be reached on hardware and software once information governance had been properly defined. The synergies resulting from integrating information systems from different organizations then positively affected service quality. In another study, health and social care managers also acknowledged that undertaking considerable work together to agree on what could be shared helped to implement a shared electronic record between nursing and adult social care practitioners [10]. While fostering cross-organizational relationships was important, substantial resources were required to develop and sustain these relationships [49].

#### Digital Readiness and Organizational Capacity for Change

The importance of investing in the necessary groundwork and anticipating the work involved in digital implementation is linked to an organization’s digital readiness and capacity for new technology more generally. Facilitators and barriers within this theme were related to hardware and internet connectivity issues, funding issues in the sector, organizational infrastructure, and resourcing the work required for digital change, including leadership and training.

##### Hardware and Internet Connectivity Issues

Hardware issues hindered implementation in 4 studies [6,8,11,55] and negatively impacted care quality in 2 studies [6,11]. Problems included a lack of computers and handheld devices for timely patient data documentation in nursing homes [6,8], ergonomic challenges in home care [55], and poor battery lives on portable devices in home care [11,55]. In Australia, residential aged care nurses and care workers relied on memory when portable devices ran out of battery during medication rounds, reducing patient safety [11]. Sharing limited devices in nursing homes also delayed access to updated care plans in a cross-country nursing home study [6]. Hardware issues implied a failure to commit the upfront investment needed to install the hardware required to successfully implement digital systems, reflecting a lack of organizational capacity and readiness [11,55]. Internet connectivity issues were identified as barriers in 5 studies [13,42,48,55,56]. Reliable internet was often deemed essential for digital implementation, and poor connectivity indicated insufficient organizational resources. This was problematic in home care, where mobile internet access was inconsistent [55,56], and in care homes with poor Wi-Fi in old buildings [13,48]. For instance, 18% of care homes in a southeast Scotland project experienced regular internet interruption, and 27% of care homes had limited internet access [42].

##### Funding Issues in the Sector

In total, 9 studies [3,9,14,16,18,48-50,56] identified insufficient funding or financial incentives and high costs as barriers. Four studies were conducted in the United States [9,14,16,18], 4 studies were conducted in England [3,48-50], and 1 study was conducted in Sweden [56]. In England, short-term funding pushed organizations toward unambitious digital solutions [3]. The financial pressures often forced providers to adopt a short-term view on the finances needed to implement and sustain digital records, constraining the scale of change and preventing it from being embedded [3]. Where funding was available, finding, requesting, and receiving it was not always straightforward [48]. Small care homes in England faced issues such as poor communication from funders, complicated application procedures, and delays in receiving funds [48].

##### Organizational Infrastructure and Resourcing the Work Required for Digital Change

Barriers related to organizational infrastructure were noted in 5 studies [19,20,48,49,56]. Issues included insufficient ICT and human resources staff [48,56], high senior staff turnover [49], poor internal communication that left staff unaware of implementation [19,20], and inadequate leadership [20]. One English study [3] highlighted that successful implementation required clear planning and resource allocation; for example, phased deployment of resources demonstrated providers’ competence in managing digital change, making it easier for them to secure further funding [3].

Four studies [3,10,50,53], 3 of which were conducted in England, noted the importance of skillful leadership in enhancing digital readiness. Identifying leaders with the right skills was crucial for managing large-scale digital projects [3]. The type of leadership required depended on context, with some providers preferring leaders who could balance risk and reward in deploying resources, while others sought leaders who were respected by their peers to help foster engagement among staff [3]. Senior staff functioning as “change agents” also motivated practitioners to review their practices [10]. Successful councils had strong leadership support for digital initiatives [50]. In England, councils successful in implementing data standards and interoperability had strong leadership support [50], with directors of social care, chief information officers, and elected members all prioritizing digital working and integrated care [50].

##### Adequate Training

Absent or inadequate training was a barrier in 4 studies [6,19,20,48]. Issues included a lack of tailored training [20] and inappropriate content [19]. Conversely, 5 studies identified high-quality training as a facilitator [3,6,11,49,53]. One multicountry study identified both facilitators and barriers across the different contexts [6]. Effective training was tailored to practitioners’ skills and tasks [6,53] and included on-the-job and context-specific training [6], ongoing sessions [55], follow-up visits [53], and continued onsite support from suppliers [6,11]. High-quality training that was tailored, targeted, and practical aligned care practices with the new practices required by digital systems.

#### Using and Sharing Recorded Information Within Technical Constraints

This theme included issues with technical interoperability of digital systems, their level of usability and user-friendliness, and the extent to which they had been appropriately adapted for social care from other settings, which were often acute or primary care.

##### Interoperability

Interoperability is understood as a technology’s capacity to electronically share patient information between different systems and to use the information that has been shared [59]. Lack of interoperability was identified in 10 studies as a barrier to sharing recorded information [3,6,9,15,17,42,49,50,52,54], being reported by 57% of 491 respondents in the US [15] nursing facilities with electronic health records. Care professionals and managers in Finland [52] and senior health and care leaders in England [49] also criticized information systems for not always “communicating” with each other. While providers were adopting digital solutions, these were not necessarily increasing interoperability and risked creating new data silos [15].

In some studies, interoperability barriers were attributed to the multitude of systems used by different organizations. Across 9 nursing homes in Austria [17], managers exchanged information with at least 18 other organizations, most of which were not part of the same electronic health record system. In the United States, while 95.1% (775/815) of nursing homes had electronic medical records, only 45.8% (373/815) had some capability for information exchange with other organizations. The variety and sheer number of systems used by different providers was a concern for 8 (67%) out of 12 staff members in subsequent interviews [9].

In England [50], interoperability issues presented as systems being unable to store identification data such as the NHS number. However, local authorities were often unaware, at the procurement stage, of which digital options could store such information. There was also confusion among councils and suppliers about the possibilities and limitations of NHS number tracing. This was linked to low organizational readiness and capacity, with providers not knowing which technological features they needed when choosing a system [50]. It also related to the downstream value suppliers promised providers in terms of being transparent about what their products could offer [50].

Staff in all 3 nursing homes in a multicountry study also complained that the electronic patient records lacked interoperability and options to adjust features to meet specific needs. This implied a contradiction between customizability and interoperability, with customizable systems more likely to meet care provider needs but less likely to be compatible with other systems than off-the-shelf technology [6].

##### Usability and User-Friendliness

A total of 8 studies [6,11,12,51,52,54-56] reported barriers related to this theme, 3 of which [52,54,56] were based in Nordic countries. These barriers were more closely associated with using, rather than sharing, recorded information within technical constraints.

A total of 4 studies [51,52,54,55] found problems with the system being slow, crashing, and having unscheduled downtime. Others pointed to features that made staff work routines more inefficient, such as the example from US home care nurses needing to click 22 times to get into each individual’s medical record, a cumbersome process that had to start again when they moved on to the next patient [12]. In a multicountry study, care home staff disliked being forced to enter narrative text into the electronic patient record and preferred drop-down menus [6]. An inefficient information retrieval process within an Australian electronic Health record system meant that staff in long-term care facilities had to perform lengthy manual searches to identify wound charts, with the system also failing to alert them if they were duplicating charts that already existed [11].

In 5 studies [8,11,13,20,55], the high usability and usefulness of digital systems facilitated implementation. In 3 cases [13,20,55], systems offered easy access to information, improving the immediacy of care provision and documentation. In some instances, they enhanced the accuracy of care documentation through better information visibility [13,55] or by automating tasks that were previously manual and prone to human error [11]. Digital systems with flagging features also supported resource prioritization and management decisions [13]. These facilitators aided implementation by increasing task efficiency and supporting the knowledge generated or made visible by the technology, thereby improving data accuracy and decision-making.

##### Adapting Technology From Other Settings

Barriers in 5 studies [6,20,45,54,56] were related to digital systems that had been maladapted from other settings and were consequently deemed inappropriate for social care. In England, social care workers were less likely to perceive health information exchange systems as useful compared to health care workers and experienced issues with the user interface [20]. Staff noted that the system looked unfamiliar compared to other systems they used, as the health information exchange was primarily designed for acute and primary care settings, with little consideration given to social and community services [20].

An Australian study [13] reported successful adaptation of a digital system originally designed for an acute hospital setting to a care home involving staff at all levels, residents, and their relatives that helped to make the product appropriate for the care home setting [13]. This co-designed process facilitated implementation and increased the likelihood of success.

#### Alignment Between Care Practices and Digital Recording Practices

##### Overview

Barriers related to digital systems not matching the context of use were identified in 9 studies [3,6,7,12,45,50,54-56]. These barriers referred to misalignments between care practices within the social care sector and recording practices demanded by new digital systems. They included reduced interactions between clients and practitioners, conflicts with preferred data input methods, and exacerbation of existing organizational issues. Staff perceptions of improved care quality increased the likelihood of accepting the technology.

##### Care Quality and the Relational Nature of Social Care

A total of 5 studies [6,7,9,49,52] highlighted barriers where digital systems decreased the relational nature of social care. Problems arose when care staff experienced disruption to their relational work and viewed the technology as depersonalizing care. In Finland, new information systems increased technical tasks at the expense of relational tasks performed physically close to clients [52]. In England, digital records influenced the nature of the clinical encounter for occupational therapists. By focusing on data collection and adherence to standard procedures, they reduced opportunities for building rapport with clients [7]. Concerns also existed that technology use close to clients was intrusive and reduced care quality [6,55]. Defining the problem as a preference for “high touch” over “high tech,” a US study found that 5 out of 12 nursing home leaders feared that technology might detract from the personal experience they aimed to provide [9].

Technological features, such as prescriptive data fields, also imposed work routines that prioritized clinical data and processes. In a Scottish study, data systems in care homes promoted a task-oriented culture over resident-focused care [45]. Prescripted data fields limited the recording of social and emotional activities and care provision, leading to an overly clinical focus in the data [45].

Only 1 study found that a digital system aligned well with the relational nature of social work, facilitating implementation [13]. In Australia, an aged care ecosystem that was co-designed with staff and residents allowed care workers to multitask and spend more time with residents. This saved time for staff and improved care quality, encouraging acceptance of the system [13]. Managers noted that the technology provided prompts for tasks such as repositioning residents, better aligning care with resident needs [13]. In England, 2 studies found that perceived care quality improvements increased staff acceptance of digital systems [3,20]. Demonstrating the technology’s value to different professionals helped staff “buy into” digital change [3]. Administrative staff adopted technology for time-saving benefits, while practitioners focused on its impact on care [3]. Perceived improvements to patient safety also increased the likelihood of adopting digital systems [20].

##### Preexisting Organizational Problems

A total of 2 studies [12,19] identified barriers where digital systems exacerbated preexisting organizational problems, such as the numerous communication channels in home care organizations [12]. The lack of standardization required nurses to adapt to various communication methods, for example, contacting physicians through primary care nurses or by fax [12]. They often only discovered that their request had reached doctors through changes made to patients’ medicines [12]. Rather than standardizing processes, the new digital system added more communication channels. While this issue presented as inappropriate technology, it was rooted in inefficient work routines that predated the technology’s introduction.

##### Conflicts Between Data Recording Practices and Digital Systems

A total of 4 UK-based studies [3,7,42,50] identified barriers due to conflicts between data recording practices preferred by care providers and those permitted by digital systems. The lack of systematic data collection in care homes made it difficult to capture the complexity of care for individuals with multiple conditions and high support needs [42]. Frontline practitioners preferred narrative text input, while digital systems often emphasized coded data entry [3,50]. In 1 study, social workers entering free-text information sometimes included data about third parties without consent [3]. Such issues were linked to a lack of understanding about data quality in social care [50], requiring retraining on the importance of proper data collection and recording practices [3,50]. An English study found that conflicts between recording preferences and the recording permitted by digital systems were due to a mismatch between digital care records and occupational therapy concerns [7]. Therapists had to recode their interventions to fit the system’s structure, suggesting that the technology did not align with sector needs, rather than indicating poor recording practices.

#### Differences Between What Is Expected and What Is Achievable With Digital Systems

##### Overview

The final theme related to the gap between organizational expectations and realistic achievements with digital systems. Guidance on available technology was often inadequate, and care providers lacked internal consensus about the technology’s capabilities and what they wanted to gain from implementation. Creating a shared digital vision and adopting digital systems as part of wider cultural changes facilitated implementation.

##### Guidance on the Technology Available

Insufficient guidance on available technology was a barrier in 1 study in English care homes [48]. The overwhelming number of suppliers created an “unregulated tech product maze,” making it difficult to choose the best option and avoid paying for unsuitable technology [48]. Care homes criticized NHS England’s “assured suppliers list” of DSCR suppliers, which was introduced to aid decision-making [48]. Although suppliers on the list met a set of standards, some care homes complained that suppliers did not meet their needs and requirements, while others reported poor experiences with suppliers on the list and found themselves locked into contracts despite consistent software malfunctions [48].

##### A Shared Digital Vision

Creating a shared vision for collectively understanding the technology involved building organizational consensus on its potential while remaining realistic about its limitations. A total of 4 studies [3,17,20,52] found that care provider staff disagreed about the purpose of digital systems, and awareness of potential benefits for care delivery was low. There were tensions between 2 distinct staff groups with different expectations [3]. One group represented a technical and managerial culture that often initiated digital change projects and was primarily interested in the information captured by digital systems. The other was a clinical culture that was concerned with how technology could help deliver care and was more skeptical of changes to practice that lacked certain types of evidence [3]. Managers were generally more positive about implementation but lacked awareness of some of its negative effects on employees’ work [52]. Staff anticipated unrealistic benefits and were often unaware of the technology’s value [17,20].

A total of 2 studies found facilitators to creating a shared vision [13,53]. They highlighted the importance of co-design and inclusive implementation by gathering suggestions from staff, residents, and their relatives [13] or by conducting monitoring based on staff’s feedback to system developers [53]. Involving different groups as partners in the process helped envision a digital system that benefited everyone [13]. Professionals praised comprehensive and continuous communication that helped them make sense of a new service, with information delivered through multiple channels to reach as many employees as possible, including shift workers [53].

##### Implementing Digital Change as a Cultural Change

Framing digital implementation as a cultural change program facilitated success in 3 studies [3,10,13]. In an Australian care home, co-designing the system, establishing a shared vision across the workforce, and providing training and feedback loops instigated a culture change that improved service delivery and problem-solving [13]. In England, barriers to scaling digital changes in health and social care were mitigated by treating them as part of a wider technology-supported clinical transformation program, rather than an ICT project [3], or as part of a larger cultural change program to improve administrative efficiency [10].

### Summary of the Intended and Actual Outcomes

Outcomes of digital implementation, either intended or actual, were identified in 17 studies [7-11,13,17,19,20,48,50-52,54, 55,57,58], although they were the focus of only 1 study [57]. The full details of the benefits and outcomes are provided in Table 2.

A total of 3 studies [9,17,48] identified the outcomes that participants hoped to achieve through adopting digital systems. Improved information accessibility, information sharing, and quality of care records were identified in 2 studies [17,48], making them the most frequent intended outcomes. Examples of the improved quality of records included more complete and readily available patient-related information and less documents being lost during patient transitions between different institutions [17]. Improved efficiency [17] and time savings [48] were identified as intended outcomes in 1 study.

Three studies [9,48,50], 2 of which were based in England [48,50], cited poor awareness about the benefits of digital systems for social care or concerns that they would not benefit the sector. In England, information sharing initiatives were often focused on health care and hospitals, with less attention paid to the potential benefits for councils or social care [50]. This made it difficult for social care staff and care home residents to see the benefits that digital systems could bring [48].

A total of 13 studies identified positive outcomes realized through digital record implementation [7,8,10,11,13,19, 20,50-52,55,57,58]. Improved efficiency was the most frequent actual outcome (8/13, 62%) [10,11,13,19,20,50-52], achieved through the automation of previously manual processes [11], reduced duplication of procedures [20], and the increased availability [19] and immediacy [13] of information improving decision-making and care planning. These outcomes were associated with increased staff capacity [50] and productivity [52]. Impacts on efficiency were not always clear. In 1 study [11], while automatic data entry in patient records was beneficial, the system did not completely align with work processes, and staff needed to record some data twice.

**Table 2:** The intended and actual outcomes of digital social care record implementation (N=17).

Theme			Studies, n (%)
**Intended outcomes**			
	Improved quality of data records	2 (12)	
	Improved information sharing	2 (12)	
	Improved information accessibility	2 (12)	
	Improved efficiency	1 (6)	
	Time savings	1 (6)	
	Improved care quality or planning	1 (6)	
	Improved communication or collaboration	1 (6)	
	Improved information accuracy	1 (6)	
	Space savings (less paper)	1 (6)	
**Actual outcomes**			
	Improved efficiency	8 (47)	
	Perceived time savings	7 (41)	
	Improved information accessibility	5 (29)	
	Workarounds (viewed negatively)	4 (24)	
	Improved communication or collaboration	3 (18)	
	Improved information security and risk management	3 (18)	
	Additional time burdens	3 (18)	
	Improved care quality or planning	2 (12)	
	Increased face-to-face work with patients	2 (12)	
	Improved information sharing	2 (12)	
	Improved information accuracy	2 (12)	
	Improved transparency and accountability	2 (12)	
	Increased staff or patient satisfaction	1 (6)	
	Workarounds (viewed positively)	1 (6)	
	Decreased communication or collaboration	1 (6)	
	Decreased efficiency	1 (6)	
	Decreased care quality	1 (6)	
	Decreased face-to-face work with patients	1 (6)	
	Lack of financial benefits	1 (6)	
	Rationing care documentation	1 (6)	

Perceived time savings were reported in 7 studies, although the findings were not conclusive [10,13,19,20,50,52,58]. Some studies reported staff spending less time retrieving and documenting information for decision-making [13,58] and chasing other organizations for patients’ whereabouts [50]. One study found time savings of up to 45 minutes for long-term care staff when completing medication reconciliation [19]. However, 2 studies found time savings in some areas and additional time burdens in others [10,52]. In 1 case, disagreements between managers and their staff arose regarding whether the digital system created time savings [52]. Managers and employees agreed that moving from phone calls to digital messaging had freed up staff time for other tasks [52]. However, employees felt that the new tasks, such as responding to clients through messages, required extra time. This additional time was not always recognized by management, nor were additional resources provided [52].

A total of 3 studies [8,13,19,20,52] found that digital systems made information more accessible. In one case, this enabled person-centered care, with easily accessible information on individual backgrounds helping staff to “see the person first and the diagnosis second” [13]. In another case, improved visibility of information facilitated medication tracking and therefore supported patient safety [19]. A total of 3 studies also highlighted improved communication and collaboration [10,52,55] and improved information security and risk management [51,52,58]. Electronic information sharing improved partnership working, enhancing collaboration and increasing the timeliness, efficiency, and quality of care [10,52,55]. Improved information security and risk management were linked to secure information transfer and storage [51,52,58], better client monitoring [52], and increased data accuracy [51,58].

Workarounds, identified in 5 studies [7,9,11,54,55], were the most common negative outcome. Workarounds involve the implementation, by end users, of temporary practices or behaviors to overcome the limitations of a technological system [60]. Staff developed workarounds for various reasons. These included circumventing the system to share health data with residents [9] and accessing case-based information [54]. While workarounds could be beneficial [7] and support task completion [54], they also threatened data security [54].

## Discussion

### Principal Findings

This study investigated what is known about the implementation of digital records in adult social care settings. The literature was diverse in terms of the type of digital system, setting, and use case studied. Most of the studies used a qualitative design (14/29, 48%), particularly those looking at facilitators and barriers, how digital systems affect work routines, and potential strategies to improve digital systems. Studies were most frequently based within the United Kingdom (10/29, 34%).

Most studies focused on facilitators of and barriers to digital implementation. Many facilitators and barriers were interlinked and associated with multiple NASSS framework domains, which compounded the complexity of implementing digital systems. The 5 themes we identified using the NASSS framework are particularly complex areas that require more active management and consideration when implementing DSCRs in social care contexts.

While our findings suggest that implementing digital systems is an inherently complex process, this review did identify some strategies to manage complexity, which could constitute “good practice.” In terms of digital readiness and organizational capacity for change, high-quality training was found to increase implementation success. Where training was tailored, practical, and ongoing, it helped align care practices with new practices required by the technology, thereby increasing employees’ ability and willingness to adopt and continue to use the system. Although high-quality training depended on care provider leaders anticipating the financial resources needed, it seems a worthwhile investment for successful digital implementation. This finding echoes the results from a previous scoping review, which highlighted training as a key factor influencing the use of electronic information systems [23].

Implementing digital systems as part of wider cultural change projects also addressed multifaceted complexity. An example of this was the project in which implementation was co-designed with staff [13]. This approach enabled a shared vision of the technology to be created across the care home among residents and staff at different levels. The sense of ownership this instilled addressed complexity in the adopter system domain, with all users more likely to support the technology and view it as “business as usual.” Co-design also addressed complexity in the technology domain, with the digital system more likely to align with the needs and practices of its user group. While incorporating digital implementation as part of broader transformation required significant resources, where there was sufficient organizational readiness and capacity for comprehensive rollout, implementation seemed to have greater potential for sustainability, scaling, and spread.

Complexity related to data sharing and information governance seemed to be more difficult to address. Trust and relationship building across organizations could help establish data sharing agreements at a localized level and therefore address complexity within the organization domain. However, fundamental barriers were associated with complexity around regulations and standards in the wider context domain, over which care providers had no direct control. Until there is primary or secondary legislative change, the governance and regulatory context will continue to impede cross-organizational data sharing efforts.

Although 17 studies identified intended or actual outcomes, they more often focused on identifying facilitators of and barriers to implementation. Improved efficiency, accuracy, and time savings were the most common positive outcomes realized through digital adoption, while workarounds and additional time burdens were the most frequently cited negative outcomes. Some of the positive outcomes reflect the results presented in the review by Greenstock [22], which also found efficiency and productivity to be a benefit of DSCRs. However, the limited detail in outcome reporting and variations in the extent to which different benefits are observed suggest that this topic would benefit from future research. Specifically, there seems to be a need for studies that quantify outcomes and pay greater attention to the necessary conditions for positive benefits to be realized.

### Limitations of Studies

Most studies (20/29, 69%) lacked a clear theoretical or methodological framework. This meant it was often unclear which type of digital system or record was being implemented as well as the context, setting, and use case. While studies mentioned >100 facilitators and barriers to implementation, they did not provide any objective parameters or measures to assess how they impact implementation or social care practices. This hinders a more comprehensive comparison between the barriers and facilitators.

Some digital systems were simply described as ICT, electronic digital systems or health information technology [18,55,57], or digitalization or digital change generally [3,48,52], without definitions of these terms. Some studies appeared to use the same vocabulary to describe different systems. However, this was difficult to determine as most studies (20/29, 69%) did not specify their target population clearly. Many studies also lacked detail regarding care settings and other relevant information, which limited the possibility of performing more comprehensive comparative analysis. Future studies should pay greater attention to how they report which digital systems were implemented, the target population for the system, the setting, and the roles of the professionals involved to facilitate comparisons between studies. Standardized reporting guidelines, such as the template for intervention description and replication checklist and guide [61], may facilitate describing digital projects or systems.

Of the 29 studies, only 2 (7%) included carers or service users as respondents, while most studies included staff (n=18, 62%) or managers (n=11, 38%). Future research may therefore benefit from incorporating the perspectives of people drawing on care to cover this gap in the literature.

Although studies included in our review mention the potential impacts of DSCRs, none provide quantifiable parameters to estimate such impacts, such as potential time savings or cost-effectiveness metrics. New studies that are appropriately designed to measure such outcomes are needed to fill this important knowledge gap in the literature on DSCR implementation.

### Methodological Limitations

Due to the prevalence of qualitative designs and a lack of clear theoretical or methodological frameworks among the studies reviewed, we used the NASSS framework as a structured approach to categorizing and interpreting heterogeneous data. As this was a rapid review, the framework served as a tool to guide our data interpretation and triangulation, especially given the large number of barriers and facilitators and the varied ways these issues were described across different studies. For example, it directed our analysis of hardware and internet connectivity issues. While the studies reviewed often attributed these to technological problems, the framework enabled us to trace the associated complexity back to the organization domain, with care providers lacking the awareness, readiness, and capacity to prepare for digital implementation and adopt appropriate systems.

However, the NASSS framework carried some limitations for our analysis. No facilitators or barriers were associated with the condition domain. While the framework was developed for both health and social care, the focus of this domain on comorbidities and clinical aspects of a patient’s condition may be more appropriate for health care technologies. For social care technologies, it may be more useful to approach the condition domain in terms of whether digital systems are appropriate for particular groups of clients, such as older people or people with learning disabilities, rather than specific illnesses. Alternatively, the lack of relevance of the condition domain may reflect limited attention to diversity and inclusion considerations within the studies reviewed. Another limitation of the NASSS framework was related to the final domain (ie, interaction between domains and adaptation over time). As most of the complexity we identified was multifaceted, we found it more useful to iteratively analyze the interactions between domains instead of restricting them to 1 domain. Rather than viewing complexity as belonging to separate domains, we suggest using this final domain to provide an overarching perspective of how complexity constantly intersects and interacts across domains at every stage of digital implementation.

Considering the rapid nature of this scoping review, we simplified some review procedures, such as screening and full-text assessment, which always carry the risk of missing relevant studies. To minimize such risks, all review procedures were undertaken by researchers who are experts in systematic review methods and social care research. As with every review, the choice of databases is also a limitation, as relevant studies may have been uniquely indexed in databases that were not included. However, our research was able to identify all relevant studies that were suggested by experts in the field. Moreover, we performed a comprehensive gray literature search to reduce the likelihood of missing key studies.

Despite the limitations, we believe that our review provides a comprehensive picture of the state of the literature on DSCRs. It builds on 4 previous reviews, which, when taken together, captured the literature about digital records until the end of 2017 [2,21-23]. Our review has updated and added to these findings, covering both academic and gray literature up until 2023 and using a robust theoretical framework to draw out complexity in terms of sustainability, scaling, spread, nonadoption, and abandonment of digital care records.

### Conclusions

Our findings suggest that the implementation of digital care records is particularly complex due to the lack of a common language and consensus about what DSCRs should look like as well as expected outcomes and impacts. This is reflected in the scientific literature, which often lacks operationalization of key constructs and robust study designs. To be successful, implementation should consider complexity, while studies should use a robust theoretical framework and use mixed methods or quantitative designs where appropriate. We also suggest that future studies define the target population, consider gathering data on the experiences of carers and service users, and focus on digital care records specifically being used in social care, such as DSCRs.

## Appendix

Multimedia Appendix 1 PRISMA-ScR (Preferred Reporting Items for Systematic Reviews and Meta-Analyses extension for Scoping Reviews) checklist.

Multimedia Appendix 2 Search Strategy, August 14, 2023.

Multimedia Appendix 3 Detailed characteristics of the included studies (N=29).

Multimedia Appendix 4 Thematic analysis of the facilitators and barriers using the nonadoption, abandonment, scale-up, spread, and sustainability framework.

## Abbreviations

DSCR: digital social care record
ICT: information communication technology
NASSS: nonadoption, abandonment, scale-up, spread, and sustainability
NHS: National Health Service
PRISMA-S: Preferred Reporting Items for Systematic reviews and Meta-Analyses literature search extension
PRISMA-ScR: Preferred Reporting Items for Systematic Reviews and Meta-Analyses extension for Scoping Reviews

## Data Availability

No new data were generated for this scoping review. All data were obtained from publicly available published sources and are included in this published article and its supplementary information files. We have included the search strategy in Multimedia Appendix 2, summaries of the extracted articles in Multimedia Appendix 3, and the thematic analysis process in Multimedia Appendix 4 to provide transparency on extractions and analysis.
