# Supplementary material for: Best Practices for Implementing Electronic Care Records in Adult Social Care: Rapid Scoping Review
Source: JMIR Aging. 2025 Feb 14;8:e60107. doi: 10.2196/60107 (PMC11888009; doi:10.2196/60107)
Supplement: Multimedia Appendix 2 [file aging_v8i1e60107_app2.docx]

# Multimedia Appendix 2: Search Strategy, August 14, 2023

Contents

[Multimedia Appendix 1: Search Strategy, August 14, 2023 1](#_Toc1706952331)

[MEDLINE 1](#_Toc979190385)

[Emcare 2](#_Toc1152479891)

[Web of Science 3](#_Toc727499209)

[HMIC Health Management Information Consortium <1979 to May 2023> 4](#_Toc1730016655)

[Social Policy and Practice <202304> 5](#_Toc360586211)

[Social Services Abstracts 5](#_Toc1459618841)

[Search Information 6](#_Toc752151431)

[Authors 6](#_Toc1627839728)

## MEDLINE

Ovid MEDLINE(R) ALL <1946 to August 02, 2023>

1 ((electronic* or online or on-line or digital* or platform* or solution* or portal*) adj4 (((personal or care or case or client or service-user or service user or resident) adj2 (record* or data or system*)) or (care home adj2 data) or data set*)).ti,ab,kf. 3061

2 ((web or internet or computer* or information system* or (information adj2 technology) or ICT or (communication adj2 technology)) adj3 (((personal or care or case or client or service-user or service user or resident) adj2 (record* or data or system*)) or (care home adj2 data) or data set*)).ti,ab,kf. 1815

3 dscr.ti,ab,kf. 103

4 1 or 2 or 3 4895

5 exp Social Support/ 79873

6 exp Social Work/ 18653

7 exp Residential Facilities/ 58375

8 exp Long-Term Care/ 28478

9 exp Home Care Services/ 50878

10 ((social adj3 care) or (social adj3 service*) or social supports or (disab* adj3 care) or (social adj3 work*) or (adult adj3 service*) or (residential adj3 care) or (residential adj3 facilit*) or (home adj3 care) or (domiciliary adj3 care) or (care adj3 home*) or (nursing adj3 home*) or (nursing adj3 facilit*)).ti,ab,kf. 146524

11 (long term care or longterm care or LTC or ((aged or aging or elder* or old* people or old* adult) adj3 care)).ti,ab,kf,kw. 50992

12 ((Respite or special needs or Rehabilitation or Dementia or End-of-life or "mental health" or Personal or Welfare or Community or "Aging services" or "Senior housing" or Homelessness or Addiction or Hospice or "Assisted living" or "Aging in place" or "Continuing care retirement community" or CCRC) adj3 care).ti,ab,kf. 89656

13 5 or 6 or 7 or 8 or 9 or 10 or 11 or 12 392014

14 ((Interoperability or (Information adj2 sharing) or (data adj2 sharing) or (Information adj2 exchange) or (Information adj2 flow) or (Information adj2 access)) and (electronic* or online or on-line or digital* or platform* or solution* or portal* or web or internet or computer* or information system* or (information adj2 technology) or ICT)).ti,ab,kf. 15520

15 4 and 13 453

16 limit 15 to (english language and yr="2018 - 2024") **186**

17 13 and 14 1002

18 limit 17 to (english language and yr="2018 - 2024") **439**

19 16 or 18 **610**

## Emcare

Ovid Emcare <1995 to 2023 Week 31>
Searched 7/8/23

1 ((electronic* or online or on-line or digital* or platform* or solution* or portal*) adj4 (((personal or care or case or client or service-user or service user or resident) adj2 (record* or data or system*)) or (care home adj2 data) or data set*)).ti,ab,kf. 1876

2 ((web or internet or computer* or information system* or (information adj2 technology) or ICT or (communication adj2 technology)) adj3 (((personal or care or case or client or service-user or service user or resident) adj2 (record* or data or system*)) or (care home adj2 data) or data set*)).ti,ab,kf. 1009

3 dscr.ti,ab,kf. 20

4 1 or 2 or 3 2857

5 exp social support/ 48201

6 exp social work/ 19857

7 exp residential home/ 3093

8 exp long term care/ 411710

9 exp home care/ 32374

10 ((social adj3 care) or (social adj3 service*) or social supports or (disab* adj3 care) or (social adj3 work*) or (adult adj3 service*) or (residential adj3 care) or (residential adj3 facilit*) or (home adj3 care) or (domiciliary adj3 care) or (care adj3 home*) or (nursing adj3 home*) or (nursing adj3 facilit*)).ti,ab,kf. 114509

11 (long term care or longterm care or LTC or ((aged or aging or elder* or old* people or old* adult) adj3 care)).ti,ab,kf,kw. 35525

12 ((Respite or special needs or Rehabilitation or Dementia or End-of-life or "mental health" or Personal or Welfare or Community or "Aging services" or "Senior housing" or Homelessness or Addiction or Hospice or "Assisted living" or "Aging in place" or "Continuing care retirement community" or CCRC) adj3 care).ti,ab,kf. 63424

13 5 or 6 or 7 or 8 or 9 or 10 or 11 or 12 630301

14 ((Interoperability or (Information adj2 sharing) or (data adj2 sharing) or (Information adj2 exchange) or (Information adj2 flow) or (Information adj2 access)) and (electronic* or online or on-line or digital* or platform* or solution* or portal* or web or internet or computer* or information system* or (information adj2 technology) or ICT)).ti,ab,kf. 9683

15 4 and 13 459

16 limit 15 to (english language and yr="2018 - 2024") 180

17 13 and 14 947

18 limit 17 to (english language and yr="2018 - 2024") 385

19 16 or 18 **557**

## Web of Science

# Database: Web of Science Core Collection

# Entitlements:

- WOS.SCI: 1900 to 2023

- WOS.AHCI: 1975 to 2023

- WOS.ESCI: 2015 to 2023

- WOS.ISTP: 1990 to 2023

- WOS.SSCI: 1956 to 2023

- WOS.ISSHP: 1990 to 2023

Searched 3/8/23

# Searches:

| 1. | ((electronic* or online or on-line or digital* or platform* or solution* or portal*) NEAR/4  ((personal or care or case or client or service-user or "service user" or resident) NEAR/2 (record*  or data or system*))) (Topic) OR ((web or internet or computer* or "information system*" or  (information NEAR/2 technology) or ICT or (communication NEAR/2 technology)) NEAR/3  (((personal or care or case or client or service-user or "service user" or resident) NEAR/2  (record* or data or system*)) or ("care home" NEAR/2 data) or "data set*")) (Topic) OR dscr*  (Topic) Date Run: Thu Aug 03 2023 21:27:32 GMT+0100 (British Summer Time) | Results:  14869 | Records concept |
| --- | --- | --- | --- |
| 2 | TS=(((social NEAR/3 care) or (social NEAR/3 service*) or "social supports" or (disab* NEAR/3  care) or (social NEAR/3 work*) or (adult NEAR/3 service*) or (residential NEAR/3 care) or  (residential NEAR/3 facilit*) or (home NEAR/3 care) or (domiciliary NEAR/3 care) or (care  NEAR/3 home*) or (nursing NEAR/3 home*) or (nursing NEAR/3 facilit*))) OR TS=(("long term  care" or "longterm care" or LTC or ((aged or aging or elder* or "old* people" or "old* adult")  NEAR/3 care))) OR TS=(((Respite or "special needs" or Rehabilitation or Dementia or End-of-life  or "mental health" or Personal or Welfare or Community or "Aging services" or "Senior housing"  or Homelessness or Addiction or Hospice or "Assisted living" or "Aging in place" or "Continuing  care retirement community" or CCRC) NEAR/3 care)) Date Run: Thu Aug 03 2023 21:28:44  GMT+0100 (British Summer Time) | Results: 476743 | Social care concept |
| 3 | Interoperability or (Information NEAR/2 sharing) or (data NEAR/2 sharing) or (Information  NEAR/2 exchange) or (Information NEAR/2 flow) or (Information NEAR/2 access) (Topic) AND  electronic* or online or on-line or digital* or platform* or solution* or portal* or web or internet or  computer* or "information system*" or (information NEAR/2 technology) or ICT (Topic) Date  Run: Thu Aug 03 2023 21:34:34 GMT+0100 (British Summer Time) | Results: 98588 | Interoperablity concept |
| 4 | #1 and #2 Timespan: 2018-01-01 to 2024-12-30 Date Run: Thu Aug 03 2023 22:23:14  GMT+0100 (British Summer Time) | Results: 623 | Combined Records and Social Care. 2018- |
| 5 | #1 and #2 and English (Languages) Timespan: 2018-01-01 to 2024-12-30 Date Run: Thu  Aug 03 2023 22:23:42 GMT+0100 (British Summer Time) | Results: 618 | Combined Records and Social Care. 2018-. English, |
| 6 | #3 AND #2 Date Run: Thu Aug 03 2023 22:24:18 GMT+0100 (British Summer  Time) | Results: 2528 | Combined Social Care and Interoperability, |
| 7 | #3 AND #2 Timespan: 2018-01-01 to 2024-12-30 Date Run: Thu Aug 03 2023 22:24:54  GMT+0100 (British Summer Time) | Results: 1225 | Combined Social Care and Interoperability, 2018- |
| 8 | #3 AND #2 and English (Languages) Timespan: 2018-01-01 to 2024-12-30 Date Run:  Thu Aug 03 2023 22:25:03 GMT+0100 (British Summer Time) | Results: 1199 | Combined Social Care and Interoperability, 2018- English |
| 9 | #8 OR #5 Date Run: Thu Aug 03 2023 22:26:21 GMT+0100 (British Summer  Time) | Results: 1765 | Combined Records and Social Care. 2018-. English **OR** Combined Social Care and Interoperability, 2018- English |

## HMIC Health Management Information Consortium <1979 to May 2023>

Ovid platform

Searched 03/08/23

1 ((electronic* or online or on-line or digital* or platform* or solution* or portal*) adj4 (((personal or care or case or client or service-user or service user or resident) adj2 (record* or data or system* or information)) or (care home adj2 data) or data set*)).mp. 172

2 ((web or internet or computer* or information system* or (information adj2 technology) or ICT or (communication adj2 technology)) adj3 (((personal or care or case or client or service-user or service user or resident) adj2 (record* or data or system*)) or (care home adj2 data) or data set*)).mp. 155

3 dscr.mp. 0

4 1 or 2 or 3 325

5 ((social adj3 care) or (social adj3 service*) or social supports or (disab* adj3 care) or (social adj3 work*) or (adult adj3 service*) or (residential adj3 care) or (residential adj3 facilit*) or (home adj3 care) or (domiciliary adj3 care) or (care adj3 home*) or (nursing adj3 home*) or (nursing adj3 facilit*)).mp. 49062

6 (long term care or longterm care or LTC or ((aged or aging or elder* or old* people or old* adult) adj3 care)).mp. 6788

7 ((Respite or special needs or Rehabilitation or Dementia or End-of-life or "mental health" or Personal or Welfare or Community or "Aging services" or "Senior housing" or Homelessness or Addiction or Hospice or "Assisted living" or "Aging in place" or "Continuing care retirement community" or CCRC) adj3 care).mp. 21316

8 5 or 6 or 7 65233

9 ((Interoperability or (Information adj2 sharing) or (data adj2 sharing) or (Information adj2 exchange) or (Information adj2 flow) or (Information adj2 access)) and (electronic* or online or on-line or digital* or platform* or solution* or portal* or web or internet or computer* or information system* or (information adj2 technology) or ICT)).mp. 1442

10 4 and 8 83

11 8 and 9 207

12 10 or 11 280

13 limit 12 to yr="2018 - 2023" 37

## Social Policy and Practice <202304>

Ovid platform
Searched 03/08/23

1 ((electronic* or online or on-line or digital* or platform* or solution* or portal*) adj4 (((personal or care or case or client or service-user or service user or resident) adj2 (record* or data or system* or information)) or (care home adj2 data) or data set*)).mp. 121

2 ((web or internet or computer* or information system* or (information adj2 technology) or ICT or (communication adj2 technology)) adj3 (((personal or care or case or client or service-user or service user or resident) adj2 (record* or data or system*)) or (care home adj2 data) or data set*)).mp. 57

3 dscr.mp. 0

4 1 or 2 or 3 176

5 ((social adj3 care) or (social adj3 service*) or social supports or (disab* adj3 care) or (social adj3 work*) or (adult adj3 service*) or (residential adj3 care) or (residential adj3 facilit*) or (home adj3 care) or (domiciliary adj3 care) or (care adj3 home*) or (nursing adj3 home*) or (nursing adj3 facilit*)).mp. 134941

6 (long term care or longterm care or LTC or ((aged or aging or elder* or old* people or old* adult) adj3 care)).mp. 14385

7 ((Respite or special needs or Rehabilitation or Dementia or End-of-life or "mental health" or Personal or Welfare or Community or "Aging services" or "Senior housing" or Homelessness or Addiction or Hospice or "Assisted living" or "Aging in place" or "Continuing care retirement community" or CCRC) adj3 care).mp. 31506

8 5 or 6 or 7 150478

9 ((Interoperability or (Information adj2 sharing) or (data adj2 sharing) or (Information adj2 exchange) or (Information adj2 flow) or (Information adj2 access)) and (electronic* or online or on-line or digital* or platform* or solution* or portal* or web or internet or computer* or information system* or (information adj2 technology) or ICT)).mp. 1420

10 4 and 8 128

11 8 and 9 655

12 10 or 11 753

13 limit 12 to yr="2018 - 2023" 179

## Social Services Abstracts

ProQuest

Searched 11-08-23

Set#: S1

Searched for: ((electronic* or online or on-line or digital* or platform* or solution* or portal*) NEAR/4 ((personal or care or case or client or service-user or "service user" or resident) NEAR/2 (record* or data or system*))) OR ((web or internet or computer* or "information system*" or (information NEAR/2 technology) or ICT or (communication NEAR/2 technology)) NEAR/3 (((personal or care or case or client or service-user or "service user" or resident) NEAR/2 (record* or data or system*)) or ("care home" NEAR/2 data) or "data set*")) OR dscr*

Databases: Social Services Abstracts

Results: 843

Set#: S2

Searched for: (((social NEAR/3 care) or (social NEAR/3 service*) or "social supports" or (disab* NEAR/3 care) or (social NEAR/3 work*) or (adult NEAR/3 service*) or (residential NEAR/3 care) or (residential NEAR/3 facilit*) or (home NEAR/3 care) or (domiciliary NEAR/3 care) or (care NEAR/3 home*) or (nursing NEAR/3 home*) or (nursing NEAR/3 facilit*))) OR (("long term care" or "longterm care" or LTC or ((aged or aging or elder* or "old* people" or "old* adult") NEAR/3 care))) OR (((Respite or "special needs" or Rehabilitation or Dementia or End-of-life or "mental health" or Personal or Welfare or Community or "Aging services" or "Senior housing" or Homelessness or Addiction or Hospice or "Assisted living" or "Aging in place" or "Continuing care retirement community" or CCRC) NEAR/3 care))

Databases: Social Services Abstracts

Results: 372026

Set#: S3

Searched for: Interoperability or (Information NEAR/2 sharing) or (data NEAR/2 sharing) or (Information NEAR/2 exchange) or (Information NEAR/2 flow) or (Information NEAR/2 access) AND electronic* or online or on-line or digital* or platform* or solution* or portal* or web or internet or computer* or "information system*" or (information NEAR/2 technology) or ICT

Databases: Social Services Abstracts

Results: 75071

Set#: S4

Searched for: [S1] AND [S2]

Databases: Social Services Abstracts

These databases are searched for part of your query.

Results: 745

Set#: S6

Searched for: ([S1] AND [S2]) AND (la.exact("ENG") AND pd(20180101-20241230))

Databases: Social Services Abstracts

These databases are searched for part of your query.

Results: 318

## Search Information

The search was undertaken between 02/08/23-11/08/23 by two health librarians (KP & SDG) on MEDLINE (through Ovid; KP); EmCare (through Ovid; SDG); Web of Science Core Collection (Clarivate; KP); HMIC Health Management Information Consortium (through Ovid; KP); Social Policy and Practice (through Ovid; KP); and Social Services Abstracts (through ProQuest; SDG).

## Authors

Karen Poole (KP)
Clinical Support Librarian, Libraries & Collections, King’s College London, London, UK

Sonya Di Giorgio (SDG)
Head of Clinical Library Services, Libraries & Collections, King’s College London, London, UK
