# Supplementary material for: Best Practices for Implementing Electronic Care Records in Adult Social Care: Rapid Scoping Review
Source: JMIR Aging. 2025 Feb 14;8:e60107. doi: 10.2196/60107 (PMC11888009; doi:10.2196/60107)
Supplement: Multimedia Appendix 3 [file aging_v8i1e60107_app3.docx]

# Multimedia Appendix 3: Detailed characteristics of included studies (N=29).

| **Author, year** | **Publication type** | **Country** | **Setting** | **Population** | **Aim category** | **Digital system** | **Methods** | **Participants** |
| --- | --- | --- | --- | --- | --- | --- | --- | --- |
| Aird et al, 2022 | Peer-reviewed | Canada | Hospital and long-term care home | Patients moving from emergency department to long-term care home | Identification of barriers and/or facilitators to the implementation of DSCRs and/or information exchange systems | Data integration solution between an acute hospital information system (Epic) and a long-term care home electronic medical record system (PointClickCare) | Mixed methods: Analysis of digital records; structured interviews | Emergency department physicians and residents (n=33); long-term care physician (n=1); long-term care nurse practitioner (n=1) |
| Alexander et al, 2022 | Peer-reviewed | United States | Nursing homes | Nursing home residents (no further specification) | Proportion/prevalence of services using DSCR/s and/or information exchange system and/or how these are being used | Health information technology | Cross-sectional survey | Nursing homes (n=719) |
| Ausserhofer et al, 2021 | Peer-reviewed | Switzerland | Nursing homes | Not specified | To assess professionals’ perceptions about the use of DSCRs | Electronic health records | Cross-sectional survey | Care workers, defined as registered nurses and licensed practical nurses (n=1975) |
| Bail et al, 2023 | Peer-reviewed | Australia | Private residential care home | Older people in residential care home | Identification of barriers and/or facilitators to the implementation of DSCRs and/or information exchange systems | Digital care system (Aged Care Ecosystem) | Qualitative: (i) qualitative online survey; (ii) hallway interviews; (iii) focus groups | Residents or visitors (n=48); staff (n=65) |
| Bianchi & Trimigno, 2021 | Peer-reviewed | Italy | Home care services | Non-self-sufficient older people (65+) | Assessment of potential strategies to improving existing DSCRs or information exchange systems | Edotto regional information system | Qualitative: Interviews and document analysis | Health authority managers; chief information officer |
| Chester et al, 2021 | Peer-reviewed | England | Continuing health care team | Adults and older people with complex needs referred to continuing healthcare | Identification of barriers and/or facilitators to the implementation of DSCRs and/or information exchange systems | CareFirst | Mixed methods: (i) audit of case files; (ii) staff survey; (iii) in-depth interviews | Nurses (n=7); senior managers in primary health (n=1) and social care (n=1) |
| Emmer De Albuquerque Green & Dodhia, 2022 | Report | England | Small care homes | Not specified | Identification of barriers and/or facilitators to the implementation of DSCRs and/or information exchange systems | Digitalisation in general | Qualitative interviews | Care home managers (n=18); owners of small care home businesses (n=6) |
| Felix et al, 2021 | Peer-reviewed | United States | Nursing homes | Not specified | Proportion/prevalence of services using DSCR/s and/or information exchange system and/or how these are being used | Electronic health systems | Cross-sectional survey | Nursing home administrators |
| Gatawa et al, 2019 | Report | United Kingdom | Domiciliary care; residential and nursing care; supported living and live-in care | Not specified | How DSCRs affect the work of care professionals and care providers | PASSsystem | Mixed methods: Surveys; interviews; literature review; economic assessment | Care managers (n=57); Care workers (n=95) |
| Ibrahim et al, 2020 | Peer-reviewed | Canada | Home care | Not specified | To assess professionals’ perceptions about the use of DSCRs | Electronic digital systems | Mixed methods: Online survey comprising: (i) structured questions; (ii) open-ended questions | Registered nurses |
| Ingram et al, 2022 | Peer-reviewed | England | One sustainability and transformation partnership in London | Not specified | Identification of barriers and/or facilitators to the implementation of DSCRs and/or information exchange systems | Analytics (i.e., knowledge generated from the analysis of administrative data) | Qualitative: Semi-structured interviews | Health providers (n=6); health commissioners (n=4); social care commissioners (n=4); public health consultants (n=2); health and social care commissioner (n=4) |
| Johnston et al, 2022a; Johnston et al, 2022b | Report  Peer-reviewed | Scotland | Care homes | Older people in residential care home | Mapping of services’ readiness/maturity and/or care professionals’ capability to adopt DSCRs and/or information exchange systems | (i) Basic software (Microsoft Office); (ii) electronic management systems: Person Centred Software; Log my Care, StoriiCare, Ablyss Care Management System; CareSys; Access Care Planning, Care Control System, and CareDocs | Cross-sectional online survey | Care homes for older people (n=200) |
| Kaihlanen et al, 2023 | Peer-reviewed | Finland | Health centres | Recipients of health and social care services | To assess professionals’ perceptions about the use of DSCRs | Various information and communication technologies in health and social care (e.g., electronic patient/client records, laboratory/imaging information systems, electronic prescription) | Qualitative: Focus groups and interviews | registered nurses (n=8), public health nurses (n=5), practical nurses (n=7), physicians (n=4), social workers (n=2), a social counsellor (n=1) and a digital counsellor (n=1) |
| Lin & Tunalilar, 2022 | Peer-reviewed | United States | Assisted living communities | Adults with long-term care needs | Proportion/prevalence of services using DSCR/s and/or information exchange system and/or how these are being used | Electronic health records and health information exchange systems | National quantitative survey | Assisted living communities |
| Local Government Association, 2019 | Report | England | Councils | Not reported | Identification of barriers and/or facilitators to the implementation of DSCRs and/or information exchange systems | Digital social care records and/or information sharing platform/procedures | Qualitative: Interviews and focus groups | Council representatives not specified (n=36); vendors currently supplying systems t social care within councils (n=6) and interoperability platform provider (n=1) |
| Maguire et al, 2018 | Report | England | Health and social care organisations (n=18); GP practices (n=135) | Not specified | Identification of barriers and/or facilitators to the implementation of DSCRs and/or information exchange systems | CareCentric | Qualitative semi-structured interviews | "chief executives, chief clinical information officers, chief information officers, commissioners, change managers (people with programme management responsibilities) and frontline staff [and] national policy-makers" |
| Nadav et al, 2021 | Peer-reviewed | Finland | Health centres | Not specified | Identification of barriers and/or facilitators to the implementation of DSCRs and/or information exchange systems | Digital services (e.g., digital symptom questionnaires, self-management instructions, and remote health care appointments) | Qualitative focus groups | Registered nurses (n=8); public health nurses (n=5); practical nurses (n=7); physicians (n=5); social workers (n=3); social counsellor (n=1); digital counsellor (n=1) |
| Persson et al, 2023 | Peer-reviewed | Sweden | Swedish home care organisations (n=4) | Older people living in their own homes and receiving care from nurses | Mapping of services’ readiness/maturity and/or care professionals’ capability to adopt DSCRs and/or information exchange systems | Digital tools, including electronic health records | Qualitative semi-structured interviews | Home care nurses (n=24); nurse practitioner (n=1); home care nursing strategist (n=1) |
| Powell et al, 2021 | Peer-reviewed | United States | Nursing homes | Not specified | Mapping of services’ readiness/maturity and/or care professionals’ capability to adopt DSCRs and/or information exchange systems | Computerized medical records and health information exchange | Mixed methods: Secondary analysis of data from national survey; structured interviews; qualitative interviews | Nursing home administrative leaders (n=815); nursing home administrators (n=12) |
| Qian et al, 2019 | Peer-reviewed | Australia | Care units (n=8) in long-term care facilities (n=2) | Older people in long-term care facilities | How DSCRs affect the work of care professionals and care providers | Electronic health record systems and electronic medication administration record systems | Qualitative: ethnography | Registered nurses (n=2); endorsed enrolled nurses (n=5); personal care workers (n=4) |
| Rydenfält et al, 2019 | Peer-reviewed | Sweden | Home care nursing | Not specified | Proportion/prevalence of services using DSCR/s and/or information exchange system and/or how these are being used | e-Health services | Qualitative survey with open-ended questions | Not specified |
| Salovaara & Ylonen, 2022 | Peer-reviewed | Finland | Municipal social services | Not specified | How DSCRs affect the work of care professionals and care providers | Various client information systems | Mixed methods: Online survey comprising quantitative and qualitative questions | Social workers (n=309) |
| Schaller et al, 2020 | Book chapter | Austria | Nursing homes | Not specified | Proportion/prevalence of services using DSCR/s and/or information exchange system and/or how these are being used | Electronic health record systems | Qualitative semi-structured interviews | Nursing managers (n=9) |
| Shenkin et al, 2022; Johnston et al, 2020 | Peer-reviewed  Pre-print | Scotland | Care homes | Not specified | Proportion/prevalence of services using DSCR/s and/or information exchange system and/or how these are being used | Paper records; Person Centered Sofware; Caresys; iCareHealth | Mixed methods: (i) review of residents’ records; (ii) qualitative interviews | Care home managers (n=7) |
| Shiells et al, 2020 | Peer-reviewed | Belgium; Czech Republic; Spain | Nursing homes | People with dementia in care homes | To assess professionals’ perceptions about the use of DSCRs | Electronic patient records | Qualitative: Contextual inquire; semi-structured interviews | Occupational therapists (n=2); nurses (n=2); social workers (n=2); nurse supervisors (n=6); auxiliary nurses (n=3); auxiliary nurse supervisor (n=1); physiotherapist (n=1); art therapist (n=1); home manager (n=1); care quality manager (n=1); social care supervisor (n=1) |
| Sugarhood, 2018 | Conference abstract | England | Community health and social care services | Not specified | How DSCRs affect the work of care professionals and care providers | Digital care record systems | Qualitative: ethnography | Occupational therapists (n=14) |
| Vest et al, 2019 | Peer-reviewed | United States | Nursing facilities | Residents of care homes | Proportion/prevalence of services using DSCR/s and/or information exchange system and/or how these are being used | Electronic health records | Cross-sectional survey | Not specified |
| Watkinson et al, 2021 | Peer-reviewed | England | primary care (n=24); hospital (n=44); social care services (n=8); mental health facility (n=9); third sector (n=2); community care services (n=18) | Not specified | Identification of barriers and/or facilitators to the implementation of DSCRs and/or information exchange systems | Cerner’s health information exchange platform | Mixed methods: (i) online survey; (ii) online quantitative interviews | doctors (n=37); nurses (n=20); midwife (n=1); change managers (n=2); pharmacists (n=13); allied health professional (n=19); administrative/clerical (n=1); practice managers (n=4); other (n=8) |
| Yoshimoto et al, 2022 | Peer-reviewed | Japan | Home care services | Patients 65 years or over receiving home care services | Impact of use of information communication technology on health outcomes | Information communication technology | Retrospective cohort using digital records | 554 patients receiving home care services |

## List of references

Aird T, Holditch C, Culgin S, Vanderheyden M, Rutledge G, Encinareal C, et al. An analysis of a novel Canadian pilot health information exchange to improve transitions between hospital and long-term care/skilled nursing facility. JOURNAL OF INTEGRATED CARE. 2022;30(4):399-412. doi: 10.1108/JICA-03-2022-0022.

Alexander GL, Liu J, Powell KR, Stone PW. Examining Structural Disparities in US Nursing Homes: National Survey of Health Information Technology Maturity. JMIR aging. 2022;5(3):e37482. doi: https://dx.doi.org/10.2196/37482.

Ausserhofer D, Favez L, Simon M, Zuniga F. Electronic Health Record Use in Swiss Nursing Homes and Its Association With Implicit Rationing of Nursing Care Documentation: Multicenter Cross-sectional Survey Study. JMIR MEDICAL INFORMATICS. 2021;9(3). doi: 10.2196/22974.

Bail K, Gibson D, Hind A, Strickland K, Paterson C, Merrick E, et al. 'It enables the carers to see the person first': Qualitative evaluation of point-of-care digital management system in residential aged care. Journal of clinical nursing. 2023;32(1-2):174-90. doi: https://dx.doi.org/10.1111/jocn.16285.

Bianchi P, Trimigno M. How does information system success come about in inter-organizational networks of public services? PUBLIC MONEY & MANAGEMENT. 2021;41(3):236-45. doi: 10.1080/09540962.2019.1665361.

Chester H, Hughes J, Bowns I, Abendstern M, Davies S, Challis D. Electronic information sharing between nursing and adult social care practitioners in separate locations: a mixed-methods case study. Journal of Long-Term Care. 2021:1-11.

Emmer De Albuquerque Green C., Dodhia, P. Supporting small care home providers and their managers on their journey towards digitalisation. London: NIHR Policy Research Unit in Health and Social Care Workforce, The Policy Institute, King's College London, 2022.

Felix H, Dayama N, Morris ME, Pradhan R, Bradway C. Organizational Characteristics and the Adoption of Electronic Health Records Among Nursing Homes in One Southern State. Journal of applied gerontology : the official journal of the Southern Gerontological Society. 2021;40(5):481-8. doi: https://dx.doi.org/10.1177/0733464820906685.

Gatawa T, Swift N, Gibson S, Lindsay-Walters F. Improving social care through digital care planning: an evaluation of the PASSsystem. 2019:160-.

Ibrahim S, Donelle L, Regan S, Sidani S. A Qualitative Content Analysis of Nurses' Comfort and Employment of Workarounds With Electronic Documentation Systems in Home Care Practice. CANADIAN JOURNAL OF NURSING RESEARCH. 2020;52(1):31-44. doi: 10.1177/0844562119855509.

Ingram E, Cooper S, Beardon S, Körner K, McDonald H, Hogarth S, et al. Barriers and facilitators of use of analytics for strategic health and care decision-making: a qualitative study of senior health and care leaders’ perspectives. BMJ Open. 2022;12(2):e055504. doi: 10.1136/bmjopen-2021-055504.

Johnston L, Hockley J, Henderson D, Shenkin S. The Development of a Care Home Data Platform in Scotland: Insights from the Care Home Innovation Partnership, Lothian. medRxiv. 2020:2020.08.17.20176503. doi: 10.1101/2020.08.17.20176503.

Johnston L, Koikkalainen H, Anderson L, Lapok P, Lawson A, Shenkin SD. Foundation Level Barriers to the Widespread Adoption of Digital Solutions by Care Homes: Insights from Three Scottish Studies. International journal of environmental research and public health. 2022a;19(12). doi: https://dx.doi.org/10.3390/ijerph19127407.

Johnston L, Kokkalainen H, Anderson L, Lapok P, Lowson A, Shenkin S. Digital and data readiness of care homes for older people in South East Scotland. Edinburgh: Edinburgh Napier University, 2022b.

Kaihlanen A-M, Laukka E, Nadav J, Narvanen J, Saukkonen P, Koivisto J, et al. The effects of digitalisation on health and social care work: a qualitative descriptive study of the perceptions of professionals and managers. BMC health services research. 2023;23(1):714-. doi: https://dx.doi.org/10.1186/s12913-023-09730-y.

Lin SC, Tunalilar O. Rapid adoption of electronic health record and health information exchange among assisted living communities, 2010-2018. Journal of the American Medical Informatics Association : JAMIA. 2022;29(5):953-7. doi: https://dx.doi.org/10.1093/jamia/ocac021.

Local Government Association. Local government social care data standards and interoperability. London: 2019.

Maguire D, Evans H, Honeyman M, Omojomolo D. Digital change in health and social care. 2018:87-.

Nadav J, Anu-Marja K, Kujala S, Laukka E, Hilama P, Koivisto J, et al. How to Implement Digital Services in a Way That They Integrate Into Routine Work: Qualitative Interview Study Among Health and Social Care Professionals. Journal of Medical Internet Research. 2021:e31668.

Persson J, Larsson R, Erlingsdottir G, Rydenfalt C. How Digital Systems Are Used in Swedish Home Care Nursing Practice: A Qualitative Interview Study to Identify Challenges and Opportunities. Computers, informatics, nursing : CIN. 2023. doi: https://dx.doi.org/10.1097/CIN.0000000000001006.

Powell KR, Deroche CB, Alexander GL. Health Data Sharing in US Nursing Homes: A Mixed Methods Study. Journal of the American Medical Directors Association. 2021;22(5):1052-9. doi: https://dx.doi.org/10.1016/j.jamda.2020.02.009.

Qian S, Yu P, Bhattacherjee A. Contradictions in information technology mediated work in long-term care: An activity theoretic ethnographic study. International journal of nursing studies. 2019;98:9-18. doi: https://dx.doi.org/10.1016/j.ijnurstu.2019.05.017.

Rydenfalt C, Persson J, Erlingsdottir G, Johansson G. eHealth Services in the Near and Distant Future in Swedish Home Care Nursing. CIN-COMPUTERS INFORMATICS NURSING. 2019;37(7):366-72. doi: 10.1097/CIN.0000000000000536.

Salovaara S, Ylonen K. Client information systems' support for case-based social work: experiences of Finnish social workers. NORDIC SOCIAL WORK RESEARCH. 2022;12(3):364-78. doi: 10.1080/2156857X.2021.1999847.

Schaller M, Dornauer V, Hackl WO, Lechleitner G, Uberegger M, Ammenwerth E. Implementing National Electronic Health Records in Nursing Homes in Tyrol: A Nursing Management Perspective. Studies in health technology and informatics. 2020;271:240-7. doi: https://dx.doi.org/10.3233/SHTI200102.

Shenkin SD, Johnston L, Hockley J, Henderson DAG. Developing a care home data platform in Scotland: a mixed methods study of data routinely collected in care homes. Age and ageing. 2022;51(12). doi: https://dx.doi.org/10.1093/ageing/afac265.

Shiells K, Baquero AAD, Stepankova O, Holmerova I. Staff perspectives on the usability of electronic patient records for planning and delivering dementia care in nursing homes: a multiple case study. BMC MEDICAL INFORMATICS AND DECISION MAKING. 2020;20(1). doi: 10.1186/s12911-020-01160-8.

Sugarhood P. OCCUPATIONAL THERAPISTS AND DIGITAL CARE RECORDS: EXPLORING HEALTH AND SOCIAL CARE INTEGRATION. BRITISH JOURNAL OF OCCUPATIONAL THERAPY. 2018;81:78-.

Vest JR, Jung H-Y, Wiley Jr K, Kooreman H, Pettit L, Unruh MA. Adoption of Health Information Technology Among US Nursing Facilities. Journal of the American Medical Directors Association. 2019;20(8):995-1000.e4. doi: https://dx.doi.org/10.1016/j.jamda.2018.11.002.

Watkinson F, Dharmayat KI, Mastellos N. A mixed-method service evaluation of health information exchange in England: technology acceptance and barriers and facilitators to adoption. BMC health services research. 2021;21(1):737-. doi: https://dx.doi.org/10.1186/s12913-021-06771-z.

Yoshimoto T, Nawa N, Uemura M, Sakano T, Fujiwara T. The impact of interprofessional communication through ICT on health outcomes of older adults receiving home care in Japan – A retrospective cohort study. Journal of General and Family Medicine. 2022;23(4):233-40. doi: https://doi.org/10.1002/jgf2.534.
