# Supplementary material for: Best Practices for Implementing Electronic Care Records in Adult Social Care: Rapid Scoping Review
Source: JMIR Aging. 2025 Feb 14;8:e60107. doi: 10.2196/60107 (PMC11888009; doi:10.2196/60107)
Supplement: Multimedia Appendix 4 [file aging_v8i1e60107_app4.doc]

# Multimedia Appendix 4: Thematic analysis of facilitators and barriers using the NASSS Framework

| **NASSS domains** |
| --- |
| The organisation |
| The adopter system |
| The technology |
| The wider context |
| The value proposition |
| The interaction between domains and adaptation over time |

| **Author, year** | **Category** | **Example** | **Effect on implementation** | **NASSS sub-domain** | **Theme** |
| --- | --- | --- | --- | --- | --- |
| Aird et al, 2022 | Training | Inappropriate training content | Barrier | Organisational capacity and readiness for the technology | 4 |
|  | Awareness of implementation | Some staff unaware the technology had been implemented | Barrier |  | 2 |
| Alexander et al, 2022 | Financial resources | Less resources for nursing homes to go digital than healthcare | Barrier | Fiscal policy – Funding issues in the sector | 2 |
| Ausserhofer et al, 2021 | Hardware | Lack of sufficient computers | Barrier | Organisational capacity and readiness for the technology | 2 |
|  | Usability and user-friendliness | Increased user-friendliness linked to staff perceptions of technology’s usefulness | Facilitator | Material and technical features of the technology – usability and appropriateness | 3 |
| Bail et al, 2023 | Staff acceptance of the technology | Staff views included | Facilitator | Adoption and continued use of the technology by staff | 5 |
|  | Client acceptance of the technology | Resident-centred technology | Facilitator |  | 4 & 5 |
|  | Technology matching the context of use | Technology aided holistic view of a resident | Facilitator | Aligning care practices and recording practices | 4 |
|  | Relational nature of social care | Technology enabled staff to multitask and so spend more time with residents | Facilitator |  | 4 |
|  | Holistic vs. isolated implementation | Cultural shift occurring through implementation | Facilitator | Digital innovation as culture change | 5 |
|  | User-centred design process | Co-design | Facilitator | Building a shared vision of technology’s potential | 5 |
|  | Internet connectivity | Initial issues with internet connectivity | Barrier | Organisational capacity and readiness for the technology | 2 |
|  | Appropriateness of the technology for the sector | Technology adapted well from a clinical setting | Facilitator | Material and technical features of the technology – usability and appropriateness | 3 |
|  | Usability and user-friendliness | Flagging features supported resource prioritisation | Facilitator |  | 3 |
|  | Quality of care and/or safety | Technology aligned care more closely to resident needs and improved care quality | Facilitator | Downstream value – evidence of benefit to patients | 4 |
| Bianchi & Trimigno, 2021 | Defining governance and responsibilities | Responsibilities defined for operators collecting, loading, processing, and sending data | Facilitator | Organisational capacity and readiness for the technology | 1 |
|  | Political will and stakeholder buy-in | Local health authority and areas committed to gathering quality information about home care services | Facilitator | Wider sector/political context | 4 |
| Chester et al, 2021 | Staff acceptance of the technology | Staff views included; support at senior level increasing success | Facilitator | Adoption and continued use of the technology by staff | 2 & 5 |
|  | Holistic vs. isolated implementation | Implementation part of a larger programme to improve administrative efficiency; embedding as ‘business as usual’ | Facilitator | Digital innovation as culture change | 5 |
|  | Defining governance and responsibilities | Agencies in agreement about what could and could not be shared; responsibilities clarified | Facilitator | Organisational capacity and readiness for the technology | 1 |
| Felix et al, 2021 | Financial resources | Nursing homes lacked resources to go digital | Barrier | Fiscal policy – Funding issues in the sector | 2 |
| Gatawa et al, 2019 | Usability and user-friendliness | Time-out windows too short; slow | Barrier | Material and technical features of the technology – usability and appropriateness | 3 |
| Emmer De Albuquerque Green & Dodhia, 2022 | Staff acceptance of the technology | Fears about skills required and being tracked | Barrier | Adoption and continued use of the technology by staff | 5 |
|  | Internet connectivity | Issues with internet connectivity | Barrier | Organisational capacity and readiness for the technology | 2 |
|  | Capacity to implement the technology | Lack of infrastructure and IT staff | Barrier |  | 2 |
|  | Training | Lack of training | Barrier |  | 4 |
|  | Awareness about technology available | Small care homes lacked awareness of technology on offer | Barrier | Differences between what is expected and what is achievable with digital records | 5 |
|  | Guidance on technology available | ‘Tech maze of providers’ difficult to navigate without guidance; NHS assured suppliers list inadequate | Barrier | Downstream value – evidence of benefit to organisations | 5 |
|  | Financial resources | Sector driven by short-term funding and objectives | Barrier | Fiscal policy – funding issues in the sector | 2 |
|  | Regulation and standards | Small care homes confused about standards to adhere to | Barrier | Wider legal/regulatory context | 1 |
| Ibrahim et al, 2020 | Training | Sufficient, ongoing training and IT support | Facilitator | Organisational capacity and readiness for the technology | 4 |
|  | Internet connectivity | Issues with internet connectivity | Barrier |  | 2 |
|  | Hardware | Issues with battery life | Barrier |  | 2 |
|  | Technology matching the context of use | Misalignment of technology with staff’s workflow, tasks and preferences | Barrier | Aligning care practices and recording practices  and recording practices | 4 |
|  | Relational nature of social care | Simultaneously using technology and engaging patients decreased quality care delivery | Barrier |  | 4 |
|  | Usability and user-friendliness | Unscheduled system downtime, and the EDS slowing down and/or crashing | Barrier | Material and technical features of the technology – usability and appropriateness | 3 |
| Ingram et al, 2022 | Staff acceptance of the technology | Acceptance among leaders hindered by high senior staff turnover | Barrier | Adoption and continued use of the technology by staff | 2 |
|  | Organisational coordination required for information governance | Organisational fragmentation led to resident records stored in multiple systems | Barrier | Organisational capacity and readiness for the technology | 1 |
|  | Defining governance and responsibilities | Lack of clarity over who owned what data and how to access it | Barrier |  | 1 |
|  | Collaborating and building trust | Senior leaders built trusted relationships with analysts to access data and inform decisions | Facilitator | Interdependencies between technologies and teams | 1 |
|  |  | Reservations to share data stemmed from conflicting organisational priorities | Barrier |  | 1 |
|  | Interoperability | Data seen as disconnected in siloed systems that were unable to ‘talk to each other’ | Barrier | Sustainability issues related to interoperability of the technology | 3 |
| Johnston et al, 2022a; Johnston et al, 2022b | Defining governance and responsibilities | Lack of clarity and coordination among stakeholders | Barrier | Organisational capacity and readiness for the technology | 1 |
|  | Internet connectivity | Issues with internet connectivity | Barrier |  | 2 |
|  | Collaborating and building trust | Relationships built between providers and local authorities; ‘Data safe havens’ helped to increase willingness to share data | Facilitator | Interdependencies between technologies and teams | 1 |
|  | Interoperability | Data linking and interoperability found to be very limited currently | Barrier | Sustainability issues related to interoperability of the technology | 3 |
|  | Competition in private provider market | Private care homes concerned that sharing data could compromise their competitive advantage | Barrier | Downstream value – evidence of benefit to organisations | 1 |
|  | Regulation and standards | No established system for the governance of care home data | Barrier | Wider legal/regulatory context | 1 |
|  | Financial resources | Cost a barrier to uptake in one third of care homes (Johnston, 2022b) | Barrier | Fiscal policy – funding issues in the sector | 2 |
| Kaihlanen et al, 2023 | Staff acceptance of the technology | Senior staff lacked awareness of how implementation affects professional staff | Barrier | Adoption and continued use of the technology by staff | 5 |
|  | Communicating to reach a common understanding | Leaders communicated sufficiently with staff | Facilitator | Building a shared vision of technology’s potential | 5 |
|  | Awareness of implementation | Managers lacked awareness of impact of implementation on employees’ work | Barrier |  | 5 |
|  | Relational nature of social care | Relational work replaced by technical and information system work | Barrier | Aligning care practices and recording practices | 4 |
|  | Usability and user-friendliness | System sometimes crashed | Barrier | Material and technical features of the technology – usability and appropriateness | 3 |
|  | Interoperability | Different information systems did not always ‘communicate with each other’ | Barrier | Sustainability issues related to interoperability of the technology | 3 |
| Lin & Tunalilar, 2022 | Business case for organisations | Assisted living communities lack policy incentives to go digital | Barrier | Downstream value – evidence of benefit to organisations | 4 |
|  | Financial resources | Assisted living communities lack resources to go digital | Barrier | Fiscal policy – funding issues in the sector | 2 |
| Local Government Association, 2019 | Staff acceptance of the technology | Support at senior level increasing success, staff ownership of adoption | Facilitator | Adoption and continued use of the technology by staff | 2 & 5 |
|  | User-centred design process | Frontline staff working alongside digital teams; consultative activities | Facilitator | Building a shared vision of technology’s potential | 5 |
|  | Collaborating and building trust | Working collaboratively with other councils and suppliers | Facilitator | Interdependencies between technologies and teams | 1 |
|  |  | GPs and health practitioners unwilling to share data with social care or councils | Barrier |  | 1 |
|  | Defining governance and responsibilities | Councils/health/social care starting from different positions re information sharing | Barrier | Organisational capacity and readiness for the technology | 1 |
|  | Technology matching the context of use | Narrative recording practices needed in social care vs. data that is interoperable | Barrier | Aligning care practices and recording practices | 4 |
|  | Interoperability | Data needed for interoperability did not align with data needed for social care systems | Barrier | Sustainability issues related to interoperability of the technology | 3 |
|  | Business case for organisations | Perceived benefits small relative to cost in challenging financial climate | Barrier | Downstream value – evidence of benefit to organisations | 4 |
|  | Business case for funders | Commissioners see investment as less risky if organisations can evidence previous success with digital change | Facilitator | Downstream value – evidence of benefit to funders | 4 |
|  | Understanding/prioritising social care sector needs | Information sharing initiatives health-dominated; lack of policy attention to social care | Barrier | Wider sector/political context | 4 |
|  | Financial resources | Funding usually for siloed projects | Barrier | Fiscal policy – funding issues in the sector | 2 |
|  | Regulation and standards | Lack of data and interoperability requirements | Barrier | Wider legal/regulatory context | 1 |
| Maguire et al, 2018 | Staff acceptance of the technology | Senior level buy-in and leadership; user engagement a continual, collaborative process | Facilitator | Adoption and continued use of the technology by staff | 2 & 5 |
|  | Monitoring/evaluating technology implementation | Underinvestment in the evaluation of NHS IT projects | Barrier | Organisational resilience – adaptive and reflexive approach to implementation | 2 |
|  | Continuous improvement of system | Quality improvement initiatives kept the  momentum of digital change going | Facilitator |  | 2 |
|  | Collaborating and building trust | Engaging apprehensive staff; building trust among councils/health/social care; working together to get best deals from suppliers | Facilitator | Interdependencies between technologies and teams | 1 |
|  |  | GPs have more risk-averse attitude to sharing data | Barrier |  | 1 |
|  | Communicating to reach a common understanding | Sufficient communication between leaders and staff / users with suppliers | Facilitator | Building a shared vision of technology’s potential | 5 |
|  | Holistic vs. isolated implementation | Digital projects treated as clinical change projects | Facilitator | Digital innovation as culture change | 5 |
|  | Expectations of the technology | Managerial culture interested in digital information capture vs. clinical culture interested in how technology will assist care delivery | Barrier | Differences between what is expected and what is achievable with digital records | 5 |
|  | Technology matching the context of use | Social workers recorded third party information in free text | Barrier | Aligning care practices and recording practices | 4 |
|  | Defining governance and responsibilities | Information-sharing agreements built in from start of projects | Facilitator | Organisational capacity and readiness for the technology | 1 |
|  | Training | Training staff on technology and training digital change agents | Facilitator |  | 4 |
|  | Organisational coordination required for information governance | Sites mobilised legal and technical expertise | Facilitator |  | 1 |
|  |  | Lack of infrastructure, ‘bandwidth’, and time to implement the technology | Barrier |  | 1 |
|  | Appropriateness of the technology for the sector | Technology US-centric rather than relevant for nurses in Britain | Barrier | Material and technical features of the technology – usability and appropriateness | 3 |
|  | Interoperability | Vendor lock-in | Barrier | Sustainability issues related to interoperability of the technology | 3 |
|  | Competition in supplier market | Suppliers reluctant to open up systems to other suppliers | Barrier | Downstream value – evidence of benefit to organisations | 1 |
|  | Quality of care and/or safety | Consultants buy into technology from seeing value for patient care | Facilitator | Downstream value – evidence of benefit to patients | 4 |
|  | Financial resources | Sector driven by short-term funding and objectives | Barrier | Fiscal policy – funding issues in the sector | 2 |
|  | Regulation and standards | Lack of national guidance on data protection law | Barrier | Wider legal/regulatory context | 1 |
| Nadav et al, 2021 | Staff acceptance of the technology | Providing staff with a justification for adoption; supervisors’ attitudes toward digital services aided successful implementation | Facilitator | Adoption and continued use of the technology by staff | 2 & 5 |
|  | Monitoring/evaluating technology implementation | Usage monitoring | Facilitator | Organisational resilience – adaptive and reflexive approach to implementation | 5 |
|  | Training | Bespoke, diverse training | Facilitator | Organisational capacity and readiness for the technology | 4 |
|  | User-centred design process | Staff given influence and feedback opportunities | Facilitator | Building a shared vision of technology’s potential | 5 |
|  | Usability and user-friendliness | Absence of usability vulnerabilities | Facilitator | Material and technical features of the technology – usability and appropriateness | 3 |
| Persson et al, 2023 | Holistic vs. isolated implementation | Tasks only partially digitised; technology slotted into pre-existing problematic routines | Barrier | Digital innovation as culture change | 5 |
|  | Technology matching the context of use | Technology increased number of communication channels for nurses | Barrier | Aligning care practices and recording practices | 4 |
|  | Usability and user-friendliness | Too many clicks needed | Barrier | Material and technical features of the technology – usability and appropriateness | 3 |
| Powell et al, 2021 | Staff acceptance of the technology | Staff believed patients will increasingly expect data sharing | Facilitator | Adoption and continued use of the technology by staff | 4 |
|  | Defining governance and responsibilities | Disagreements over who owned resident data and confusion re legal basis of sharing | Barrier | Building a shared vision of technology’s potential | 5 |
|  | Collaborating and building trust | Clinical staff resistant to sharing data with nursing homes | Barrier | Interdependencies between technologies and teams | 1 |
|  | Organisational coordination required for information governance | Lack of infrastructure and time to implement the technology | Barrier | Organisational capacity and readiness for the technology | 1 |
|  | Relational nature of social care | Staff viewed technology as prioritising ‘high tech’ over ‘high touch’ care | Barrier | Aligning care practices and recording practices | 4 |
|  | Interoperability | Different systems in use | Barrier | Sustainability issues related to interoperability of the technology | 3 |
|  | Financial resources | Increased workload associated with data sharing could jeopardise already narrow profit margin | Barrier | Fiscal policy – Funding issues in the sector | 2 |
| Qian et al, 2019 | Hardware | Issues with battery life | Barrier | Organisational capacity and readiness for the technology | 2 |
|  | Training | Engineer continued as onsite resource to provide training and support | Facilitator | Organisational capacity and readiness for the technology | 4 |
|  | Usability and user-friendliness | Staff forced to do manual searches; no notification if patient data duplicated | Barrier | Material and technical features of the technology – usability and appropriateness | 3 |
|  | Appropriateness of the technology for the sector | Technology enforced the correct procedure and improved accuracy of documentation | Facilitator |  | 3 |
|  | Quality of care and/or safety | Battery issues forced staff to rely on memory in medication rounds | Barrier | Downstream value – evidence of benefit to patients | 2 |
| Rydenfält et al, 2019 | Internet connectivity | Issues with internet connectivity | Barrier | Organisational capacity and readiness for the technology | 2 |
|  | Capacity to implement the technology | Lack of HR resources to implement the technology | Barrier |  | 2 |
|  | Technology matching the context of use | Technology cannot be adjusted to the context of use | Barrier | Aligning care practices and recording practices | 4 |
|  | Usability and user-friendliness | Technology not considered mature enough for sector needs | Barrier | Material and technical features of the technology – usability and appropriateness | 3 |
|  | Appropriateness of the technology for the sector | Technology developed for contexts other than home care | Barrier |  | 3 |
| Salovaara & Ylonen, 2022 | Technology matching the context of use | Other software needed to write up care decisions as writing fields were too small in the Client Information System (CIS) | Barrier | Aligning care practices and recording practices | 4 |
|  | Appropriateness of the technology for the sector | Technology developed for health services rather than social work | Barrier | Material and technical features of the technology – usability and appropriateness | 3 |
|  | Usability and user-friendliness | Slow; patient-professional and professional-professional communications features lacked functionality | Barrier |  | 3 |
|  | Interoperability | Issues transmitting information from other systems | Barrier | Sustainability issues related to interoperability of the technology | 3 |
| Schaller et al, 2020 | Expectations of the technology | Staff hoped for benefits that the technology was unlikely to be capable of fulfilling | Barrier | Differences between what is expected and what is achievable with digital records | 5 |
|  | Interoperability | Staff needed to share information with stakeholders not on the same system | Barrier | Sustainability issues related to interoperability of the technology | 3 |
| Shenkin et al, 2022; Johnston et al, 2020 | Technology matching the context of use | Technology fuelled task-oriented culture | Barrier | Aligning care practices and recording practices | 4 |
|  | Appropriateness of the technology for the sector | Technology developed for contexts other than home care | Barrier | Material and technical features of the technology – usability and appropriateness | 3 |
| Shiells et al, 2020 | Collaborating and building trust | Fears that auxiliary nurses may not be able to use the technology correctly | Barrier | Interdependencies between technologies and teams | 1 |
|  | User-centred design process | Tailored training on the job preferred over classroom-style training | Facilitator | Building a shared vision of technology’s potential | 5 |
|  | Relational nature of social care | Staff concerned that technology was intrusive and depersonalised care | Barrier | Aligning care practices and recording practices | 4 |
|  | Technology matching the context of use | Customisability required by sector not suitable for achieving interoperability | Barrier |  | 4 |
|  | Hardware | Lack of sufficient devices | Barrier | Organisational capacity and readiness for the technology | 2 |
|  | Training | Learning ‘on the job’ would have been preferred over attending a course | Barrier |  | 4 |
|  | Interoperability | Lack of interoperable systems | Barrier | Sustainability issues related to interoperability of the technology | 3 |
|  | Appropriateness of the technology for the sector | Technology developed for services other than dementia care | Barrier | Material and technical features of the technology – usability and appropriateness | 3 |
|  | Usability and user-friendliness | Technology required narrative text when staff preferred dropdown menus | Barrier |  | 3 |
| Sugarhood, 2018 | Relational nature of social care | Lack of support from technology in developing therapeutic relationship | Barrier | Aligning care practices and recording practices | 4 |
|  | Technology matching the context of use | Technology supported information storage but did not meet other sector needs | Barrier |  | 4 |
|  | Appropriateness of the technology for the sector | Communication needs of sector did not necessarily align with the technology | Barrier | Material and technical features of the technology – usability and appropriateness | 3 |
|  | Understanding/prioritising social care sector needs | Policy voice needed for the sector to improve implementation | Barrier | Wider sector/political context | 4 |
| Vest et al, 2019 | Staff acceptance of the technology | A lack of technical know-how was a barrier to staff sharing information | Barrier | Adoption and continued use of the technology by staff | 4 |
|  | Interoperability | Staff unable to send, receive, integrate, or search for information from outside organisations | Barrier | Sustainability issues related to interoperability of the technology | 3 |
| Watkinson et al, 2021 | Staff acceptance of the technology | Inadequate communication with staff about implementation, which led to lack of expectations | Facilitator | Adoption and continued use of the technology by staff | 5 |
|  | Communicating to reach a common understanding | Leaders did not communicate about technology with staff | Barrier | Interdependencies between technologies and teams | 1 |
|  | User-centred design process | Lack of user involvement in implementation and evaluation | Barrier | Building a shared vision of technology’s potential | 5 |
|  | Training | Little to no training provided; not tailored to level of technological competence | Barrier | Organisational capacity and readiness for the technology | 4 |
|  | Defining governance and responsibilities | Unclear accountability and responsibility for success of the project | Barrier |  | 1 |
|  | Capacity to implement the technology | Lack of resources provided in social care to use the technology properly | Barrier |  | 2 |
|  | Awareness of implementation | Some staff unaware the technology had been implemented | Barrier |  | 2 |
|  | Technology matching the context of use | Technology aided holistic view of patient care | Facilitator | Aligning care practices and recording practices | 4 |
|  | Usability and user-friendliness | Real-time access to information | Facilitator | Material and technical features of the technology – usability and appropriateness | 3 |
|  | Appropriateness of the technology for the sector | Designed primarily for acute and primary care settings | Barrier |  | 3 |
|  | Quality of care and/or safety | Staff perceived quality of care to improve | Facilitator | Downstream value – evidence of benefit to patients | 4 |

## List of references

Aird T, Holditch C, Culgin S, Vanderheyden M, Rutledge G, Encinareal C, et al. An analysis of a novel Canadian pilot health information exchange to improve transitions between hospital and long-term care/skilled nursing facility. JOURNAL OF INTEGRATED CARE. 2022;30(4):399-412. doi: 10.1108/JICA-03-2022-0022.

Alexander GL, Liu J, Powell KR, Stone PW. Examining Structural Disparities in US Nursing Homes: National Survey of Health Information Technology Maturity. JMIR aging. 2022;5(3):e37482. doi: https://dx.doi.org/10.2196/37482.

Ausserhofer D, Favez L, Simon M, Zuniga F. Electronic Health Record Use in Swiss Nursing Homes and Its Association With Implicit Rationing of Nursing Care Documentation: Multicenter Cross-sectional Survey Study. JMIR MEDICAL INFORMATICS. 2021;9(3). doi: 10.2196/22974.

Bail K, Gibson D, Hind A, Strickland K, Paterson C, Merrick E, et al. 'It enables the carers to see the person first': Qualitative evaluation of point-of-care digital management system in residential aged care. Journal of clinical nursing. 2023;32(1-2):174-90. doi: https://dx.doi.org/10.1111/jocn.16285.

Bianchi P, Trimigno M. How does information system success come about in inter-organizational networks of public services? PUBLIC MONEY & MANAGEMENT. 2021;41(3):236-45. doi: 10.1080/09540962.2019.1665361.

Chester H, Hughes J, Bowns I, Abendstern M, Davies S, Challis D. Electronic information sharing between nursing and adult social care practitioners in separate locations: a mixed-methods case study. Journal of Long-Term Care. 2021:1-11.

Emmer De Albuquerque Green C., Dodhia, P. Supporting small care home providers and their managers on their journey towards digitalisation. London: NIHR Policy Research Unit in Health and Social Care Workforce, The Policy Institute, King's College London, 2022.

Felix H, Dayama N, Morris ME, Pradhan R, Bradway C. Organizational Characteristics and the Adoption of Electronic Health Records Among Nursing Homes in One Southern State. Journal of applied gerontology : the official journal of the Southern Gerontological Society. 2021;40(5):481-8. doi: https://dx.doi.org/10.1177/0733464820906685.

Gatawa T, Swift N, Gibson S, Lindsay-Walters F. Improving social care through digital care planning: an evaluation of the PASSsystem. 2019:160-.

Ibrahim S, Donelle L, Regan S, Sidani S. A Qualitative Content Analysis of Nurses' Comfort and Employment of Workarounds With Electronic Documentation Systems in Home Care Practice. CANADIAN JOURNAL OF NURSING RESEARCH. 2020;52(1):31-44. doi: 10.1177/0844562119855509.

Ingram E, Cooper S, Beardon S, Körner K, McDonald H, Hogarth S, et al. Barriers and facilitators of use of analytics for strategic health and care decision-making: a qualitative study of senior health and care leaders’ perspectives. BMJ Open. 2022;12(2):e055504. doi: 10.1136/bmjopen-2021-055504.

Johnston L, Hockley J, Henderson D, Shenkin S. The Development of a Care Home Data Platform in Scotland: Insights from the Care Home Innovation Partnership, Lothian. medRxiv. 2020:2020.08.17.20176503. doi: 10.1101/2020.08.17.20176503.

Johnston L, Koikkalainen H, Anderson L, Lapok P, Lawson A, Shenkin SD. Foundation Level Barriers to the Widespread Adoption of Digital Solutions by Care Homes: Insights from Three Scottish Studies. International journal of environmental research and public health. 2022a;19(12). doi: https://dx.doi.org/10.3390/ijerph19127407.

Johnston L, Kokkalainen H, Anderson L, Lapok P, Lowson A, Shenkin S. Digital and data readiness of care homes for older people in South East Scotland. Edinburgh: Edinburgh Napier University, 2022b.

Kaihlanen A-M, Laukka E, Nadav J, Narvanen J, Saukkonen P, Koivisto J, et al. The effects of digitalisation on health and social care work: a qualitative descriptive study of the perceptions of professionals and managers. BMC health services research. 2023;23(1):714-. doi: https://dx.doi.org/10.1186/s12913-023-09730-y.

Lin SC, Tunalilar O. Rapid adoption of electronic health record and health information exchange among assisted living communities, 2010-2018. Journal of the American Medical Informatics Association : JAMIA. 2022;29(5):953-7. doi: https://dx.doi.org/10.1093/jamia/ocac021.

Local Government Association. Local government social care data standards and interoperability. London: 2019.

Maguire D, Evans H, Honeyman M, Omojomolo D. Digital change in health and social care. 2018:87-.

Nadav J, Anu-Marja K, Kujala S, Laukka E, Hilama P, Koivisto J, et al. How to Implement Digital Services in a Way That They Integrate Into Routine Work: Qualitative Interview Study Among Health and Social Care Professionals. Journal of Medical Internet Research. 2021:e31668.

Persson J, Larsson R, Erlingsdottir G, Rydenfalt C. How Digital Systems Are Used in Swedish Home Care Nursing Practice: A Qualitative Interview Study to Identify Challenges and Opportunities. Computers, informatics, nursing : CIN. 2023. doi: https://dx.doi.org/10.1097/CIN.0000000000001006.

Powell KR, Deroche CB, Alexander GL. Health Data Sharing in US Nursing Homes: A Mixed Methods Study. Journal of the American Medical Directors Association. 2021;22(5):1052-9. doi: https://dx.doi.org/10.1016/j.jamda.2020.02.009.

Qian S, Yu P, Bhattacherjee A. Contradictions in information technology mediated work in long-term care: An activity theoretic ethnographic study. International journal of nursing studies. 2019;98:9-18. doi: https://dx.doi.org/10.1016/j.ijnurstu.2019.05.017.

Rydenfalt C, Persson J, Erlingsdottir G, Johansson G. eHealth Services in the Near and Distant Future in Swedish Home Care Nursing. CIN-COMPUTERS INFORMATICS NURSING. 2019;37(7):366-72. doi: 10.1097/CIN.0000000000000536.

Salovaara S, Ylonen K. Client information systems' support for case-based social work: experiences of Finnish social workers. NORDIC SOCIAL WORK RESEARCH. 2022;12(3):364-78. doi: 10.1080/2156857X.2021.1999847.

Schaller M, Dornauer V, Hackl WO, Lechleitner G, Uberegger M, Ammenwerth E. Implementing National Electronic Health Records in Nursing Homes in Tyrol: A Nursing Management Perspective. Studies in health technology and informatics. 2020;271:240-7. doi: https://dx.doi.org/10.3233/SHTI200102.

Shenkin SD, Johnston L, Hockley J, Henderson DAG. Developing a care home data platform in Scotland: a mixed methods study of data routinely collected in care homes. Age and ageing. 2022;51(12). doi: https://dx.doi.org/10.1093/ageing/afac265.

Shiells K, Baquero AAD, Stepankova O, Holmerova I. Staff perspectives on the usability of electronic patient records for planning and delivering dementia care in nursing homes: a multiple case study. BMC MEDICAL INFORMATICS AND DECISION MAKING. 2020;20(1). doi: 10.1186/s12911-020-01160-8.

Sugarhood P. OCCUPATIONAL THERAPISTS AND DIGITAL CARE RECORDS: EXPLORING HEALTH AND SOCIAL CARE INTEGRATION. BRITISH JOURNAL OF OCCUPATIONAL THERAPY. 2018;81:78-.

Vest JR, Jung H-Y, Wiley Jr K, Kooreman H, Pettit L, Unruh MA. Adoption of Health Information Technology Among US Nursing Facilities. Journal of the American Medical Directors Association. 2019;20(8):995-1000.e4. doi: https://dx.doi.org/10.1016/j.jamda.2018.11.002.

Watkinson F, Dharmayat KI, Mastellos N. A mixed-method service evaluation of health information exchange in England: technology acceptance and barriers and facilitators to adoption. BMC health services research. 2021;21(1):737-. doi: https://dx.doi.org/10.1186/s12913-021-06771-z.
